# miR-92a-3p regulates cisplatin-induced cancer cell death

**DOI:** 10.1038/s41419-023-06125-z

**Published:** 2023-09-13

**Authors:** Romain Larrue, Sandy Fellah, Nihad Boukrout, Corentin De Sousa, Julie Lemaire, Carolane Leboeuf, Marine Goujon, Michael Perrais, Bernard Mari, Christelle Cauffiez, Nicolas Pottier, Cynthia Van der Hauwaert

**Affiliations:** 1 University Lille, CNRS, Inserm, CHU Lille, Institut Pasteur de Lille, UMR9020-U1277 - CANTHER - Cancer Heterogeneity Plasticity and Resistance to Therapies, 59000 Lille, France; 2 Université Côte d’Azur, CNRS UMR7275, IPMC, FHU-OncoAge, IHU RespiERA, 06560 Valbonne, France

**Keywords:** Non-small-cell lung cancer, Non-coding RNAs

## Abstract

Non-small cell lung cancer is characterized by a dismal prognosis largely owing to inefficient diagnosis and tenacious drug resistance. Therefore, the identification of new molecular determinants underlying sensitivity of cancer cells to existing therapy is of particular importance to develop new effective combinatorial treatment strategy. MicroRNAs (miRNAs), a class of small non-coding RNAs, have been established as master regulators of a variety of cellular processes that play a key role in tumor initiation, progression and metastasis. This, along with their widespread deregulation in many distinct cancers, has triggered enthusiasm for miRNAs as novel therapeutic targets for cancer management, in particular in patients with refractory cancers such as those harboring KRAS mutations. In this study, we performed a loss-of-function screening approach to identify miRNAs whose silencing promotes sensitivity of lung adenocarcinoma (LUAD) cells to cisplatin. Our results showed in particular that antisense oligonucleotides directed against miR-92a-3p, a member of the oncogenic miR-17 ~ 92 cluster, caused the greatest increase in the sensitivity of KRAS-mutated LUAD cells to cisplatin. In addition, we demonstrated that this miRNA finely regulates the apoptotic threshold and the proliferative capacity of various tumor cell lines with distinct genetic alterations. Collectively, these data suggest that targeting miR-92a-3p may serve as an effective strategy to overcome treatment resistance of solid tumors.

## Introduction

Lung cancer is the leading cause of cancer-related mortality worldwide. Lung adenocarcinoma (LUAD) is the most common subtype of lung cancers and accounts for approximately 40% of all cases [1, 2]. The last 20 years have witnessed considerable progresses in the understanding of the pathogenesis of lung cancers including LUAD, with the advent of genomic technologies, the generation of several genetically engineered mouse models of lung cancers, and the construction of large databases characterizing the molecular features of respiratory tumors [3–6]. Altogether, these multidisciplinary approaches have transformed our view of LUAD from histopathological subtypes to precise molecular and genetic entities that can now be resolved at the single-cell level. Indeed, a vast array of genomic and epigenetic alterations has been reported in LUAD, in particular in genes coding for signaling proteins critical for maintaining normal cellular proliferation and survival [6–9]. Consequently, key genetic determinants are now routinely used to inform disease classification, prognostic stratification, and to support treatment decisions. For example, molecularly targeted therapies have remarkably improved treatment for patients whose tumors harbor somatically activated oncogenes such as mutant *EGFR* or translocated *ALK*, *RET* or *ROS* [10]. Nevertheless, most LUADs either lack an identifiable driver oncogene or carry mutations that are not currently clinically actionable such as those affecting *KRAS*, and are therefore still treated with conventional chemotherapy [11–13].

Despite progress in the understanding of the molecular and cellular basis and mechanisms leading to treatment failure, drug resistance continues to be the main limiting factor to achieve cures in patients with advanced cancer. Indeed, resistance of cancer cells is the major cause of tumor recurrence or relapse and affects not only conventional treatments but also targeted and immunological therapies [14, 15]. Treatment failure often results from multiple genetic and epigenetic alterations and the subsequent perturbation of critical gene and protein networks regulating tumor cell fate [16, 17]. Thus, targeting entire oncogenic networks may achieve deeper and prolonged therapeutic response than targeting individual genes or proteins within a pathway.

MicroRNAs (miRNAs), a class of small non-coding RNAs (ncRNAs) acting as negative regulators of genes involved in fundamental cellular pathways, are implicated in virtually all oncogenic processes [18, 19]. Aberrant miRNA expression is a hallmark of cancer and restoring normal miRNA levels in neoplastic cells exerts significant anti-tumor properties. The ability of miRNAs to regulate multiple genes either within a molecular oncogenic pathway or belonging to distinct but interconnected carcinogenic pathways makes them excellent drug candidates for the development of novel anti-cancer therapeutics [20]. Indeed, developing miRNA-based therapeutics is likely to be more comprehensive and efficient than targeting individual genes or proteins, especially as a limited number of miRNAs are usually dysregulated in cancer, compared to the large expression changes characterizing cancer cell transcriptome and proteome [21].

In this study, we performed a functional miRNA screening to analyze the effects of individual miRNA inhibitors on cell viability and sensitivity to cisplatin using a LUAD cell line harboring *KRAS* mutation. Moreover, our results uncovered miR-92a-3p, a member of the miR-17 ~ 92 cluster, as an important regulator of the apoptotic process in various cancer cell lines including pancreatic cancer.

## Results

### Silencing of miR-92a-3p increases sensitivity of lung adenocarcinoma cells to cisplatin

**Fig. 1:**
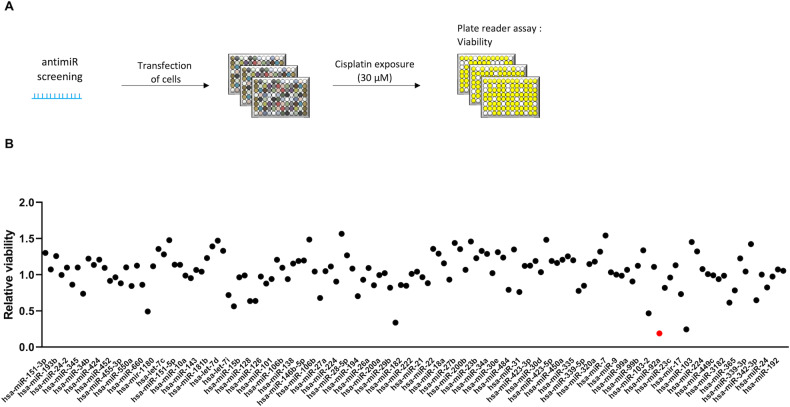
MiRNA loss-of-function screening reveals miR-92a-3p as a determinant of lung adenocarcinoma cell sensitivity to cisplatin. **A** Library of LNA-based miRNA inhibitors against the most expressed miRNAs in A549 was transfected in A549 cell line. Cells were then exposed to cisplatin at 30 µM for 72 h. Viability was measured using the CellTiterGlo assay. **B** miR-92a-3p (red dot) is the miRNA whose down-regulation is the most associated with cell death after cisplatin exposure at the indicated concentration. *n* = 2 independent experiments; ***p < 0.001.

As the most advanced miRNA-based therapeutic strategy relies on miRNA silencing, we designed a loss-of-function approach to uncover miRNAs whose decreased expression is functionally associated with increased sensitivity to cisplatin. For this, we first profiled A549 cells to identify the most highly expressed miRNAs (Supplementary Table 1), which were identified by small RNA seq, and then performed individual knockdown experiments for each of them using 138 LNA (Locked Nucleic Acid)-based anti-miRNAs (Fig. 1A). Indeed, the LUAD cell line A549 harbors KRASG12S point mutation and is widely used as a representative lung cancer cell model with KRAS mutation [22]. Our loss-of-function data identified miR-92a-3p as one of the best miRNAs whose knockdown is associated with cisplatin sensitivity (Fig. 1B).

### Modulation of miR‐92a-3p influences cisplatin-induced apoptosis of A549 cells through BIM targeting

**Fig. 2:**
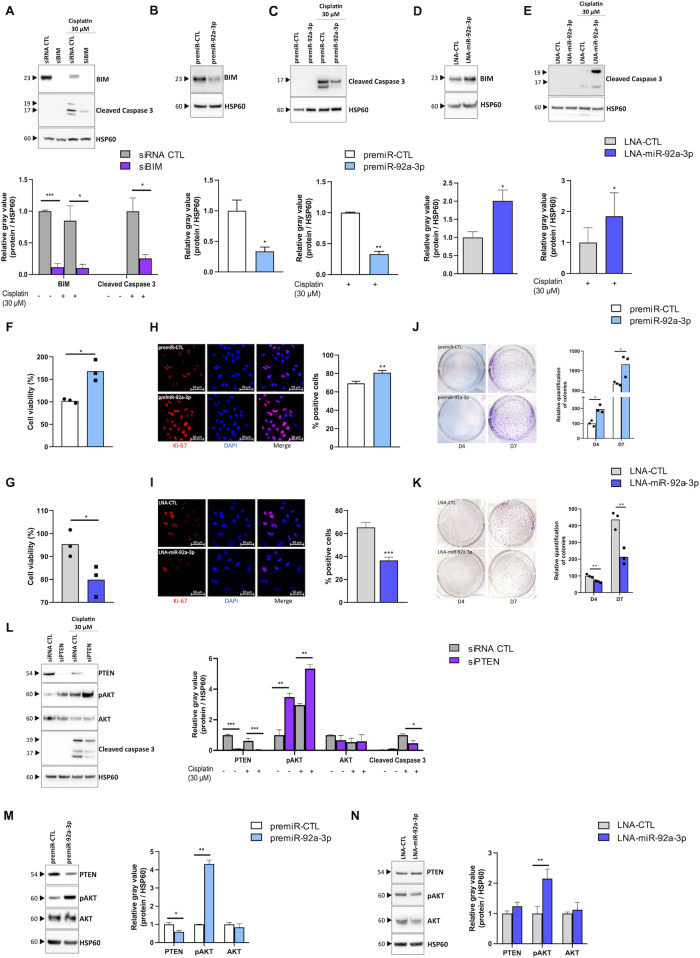
Modulation of miR-92a-3p influences the oncogenic properties of KRAS-mutated LUAD cells by targeting BIM and PTEN. **A** Western blot showing the effect of BIM inhibition on the caspase 3 apoptotic response induced by cisplatin (30 µM for 24 h). **B**–**E** Western blots showing the effect of (**B**, **D**) the modulation of miR-92a-3p on BIM expression and (**C**, **E**) on the caspase 3 apoptotic response induced by cisplatin (30 µM for 24 h). **F**, **G** Proliferation measured by cell viability 3 days after transfection of cells with (**F**) a premiR negative control and a premiR-92a-3p or (**G**) a LNA negative control and a LNA-miR-92a-3p. **H**, **I** Immunofluorescence showing the effect of (**H**) miR-92a-3p overexpression or (**I**) inhibition on Ki-67 expression. Quantitative data were obtained by measuring co-localization of DAPI staining with Ki-67 positive areas using ImageJ software. Data are presented as the mean +/− SEM. **J**, **K** Colony formation assay stained with crystal violet in cells transfected (**J**) with a premiR negative control and a premiR-92a-3p or (**K**) with a LNA negative control and a LNA-miR-92a-3p 4 and 7 days following transfection. **L** Western blot showing the effect of PTEN inhibition on the PTEN/AKT axis and on the caspase 3 apoptotic response induced by cisplatin (30 µM for 24 h). **M**, **N** Western blots showing the effect of the modulation of miR-92a-3p on the PTEN/AKT axis. Data are shown as mean +/− SEM. *n* = 3 independent experiments. Representative Western blots are shown along with their quantification. *p < 0.05; **p < 0.01; ***p < 0.001. CTL control, LNA Locked Nucleic Acid.

The BH3-only BIM protein, a bona fide target of miR-92a-3p [23], plays a major role in the initial molecular events of the intrinsic apoptotic pathway, in particular in response to anticancer drugs [24]. Indeed, tumoral expression of BIM has been shown to play a critical role in determining the response of cancer cells to not only conventional cytotoxic drugs, but also to a vast array of targeted agents [24]. Therefore, we investigated whether modulation of miR-92a-3p in LUAD cells influences their sensitivity to cisplatin through the targeting of BIM. For this, we first confirmed that siRNA-mediated silencing of BIM is sufficient to induce cisplatin resistance of LUAD cells. As depicted in Fig. 2A, siRNA-mediated silencing of BIM in A549 cells significantly reduced the cleavage of caspase 3, a reliable marker of cell apoptosis [25]. Then, we showed that modulation of miR-92a-3p inversely affects BIM expression. Indeed, overexpression of miR-92a-3p in A549 cells resulted in a strong decrease of BIM protein expression, whereas its silencing led to the induction of BIM protein expression (Fig. 2B, D). Finally, we performed gain and loss of function experiments to assess the impact of miR-92a-3p modulation on cisplatin-induced cell apoptosis. We showed that overexpression of miR-92a-3p impaired cisplatin cytotoxic effects on A549 cells, whereas its silencing exacerbated cisplatin apoptotic effects (Fig. 2C, E). As miRNAs usually regulate multiple functionally-related RNA targets to exert their function, we also investigated whether inhibiting miR-92a-3p also promotes cisplatin-induced apoptosis in BIM-depleted cells. As depicted in Fig. S1, our results show that the regulation of apoptosis by miR-92a-3p in LUAD cells is not restricted to the targeting of BIM but instead likely involves additional targets.

Altogether, these results suggest that miR-92a-3p influences LUAD cell sensitivity to cisplatin by regulating BIM expression.

### Modulation of miR‐92a-3p expression affects the proliferation of LUAD cells by targeting PTEN

To further characterize the oncogenic properties of miR-92a-3p, we investigated whether modulation of miR-92a-3p expression also impacts LUAD cell proliferation. To explore this, we first analyzed whether changes in miR-92a-3p expression affects cell growth using the CellTiterGlo cell-based assay. Our results showed that ectopic expression of miR-92a-3p in A549 cells performed three days after transfection led to an increase in cell viability that likely reflects enhanced cell growth, whereas antisense-mediated reduction of miR-92a-3p expression had the opposite effect (Fig. 2F, G). To strengthen these findings, we then performed Ki-67 staining, a reliable method for evaluating cell proliferation, using A549 cells in which miR-92a-3p is either silenced or overexpressed. As shown in Fig. 2H, I, transfection of miR-92a-3p mimics increased the number of proliferating cells compared to control cells, whereas its knockdown negatively influenced cell growth. To determine the effect of miR-92a-3p on tumor-initiating capacity, a clonogenic assay was also performed. This showed that mimic miR-92a-3p-transfected A549 cells gave rise to significantly more colonies than control cells at the indicated time points following transfection (Fig. 2J). Conversely, silencing of miR-92a-3p expression resulted in a reduced number of colonies (Fig. 2K). Finally, as miR-92a-3p has been previously reported to directly target PTEN, the main regulator of the pro-proliferative and survival PI3K/AKT signaling pathway [26, 27], we evaluated whether miR-92a-3p also influences the PTEN/AKT axis in LUAD (Fig. 2L–N). As shown in Fig. 2M, overexpression of miR-92a-3p was sufficient to reduce PTEN expression and to increase phosphorylation of AKT, whereas silencing of miR-92a-3p decreased pAKT expression (Fig. 2N). Overall, these results demonstrate that miR-92a-3p contributes to the malignant phenotype of LUAD by regulating cell proliferation.

### MiR-92a-3p also modulates the sensitivity of other LUAD cell lines with distinct molecular alterations to cisplatin

**Fig. 3:**
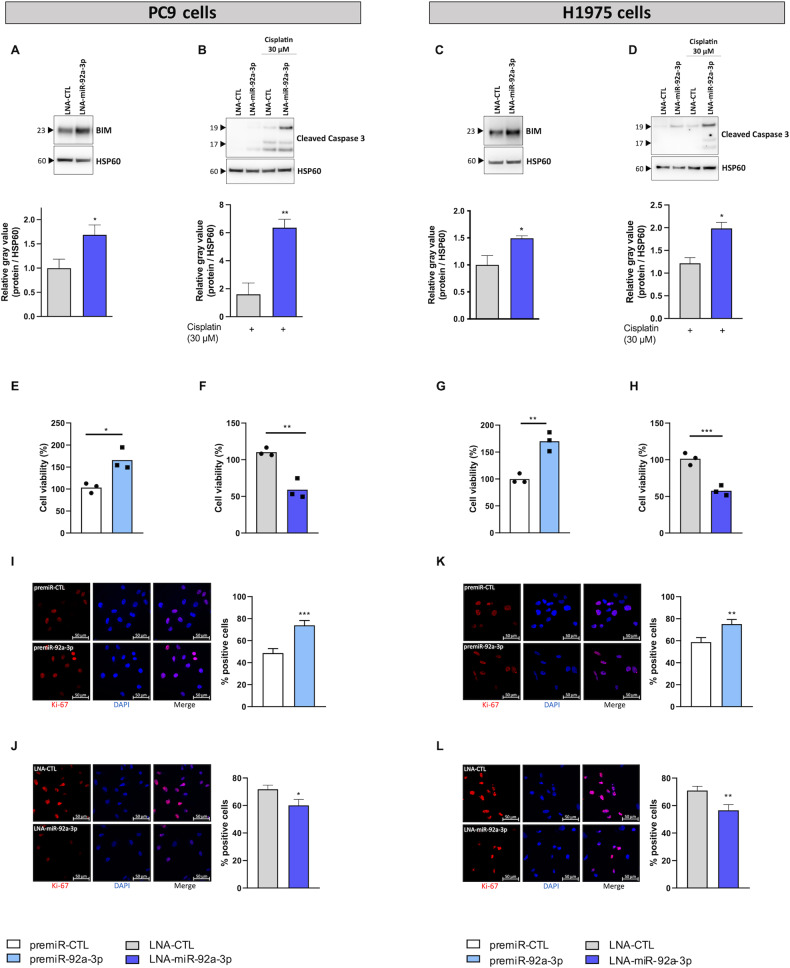
miR-92a-3p induces apoptosis in PC9 and H1975 cells in response to cisplatin exposure by targeting BIM. **A**–**D** Western blots showing the effect of the inhibition of miR-92a-3p on (**A**, **C**) BIM expression and (**B**, **D**) the caspase 3 apoptotic response induced by cisplatin (30 µM for 24 h) in both cell lines. **E**–**H** Proliferation measured by cell viability 3 days after transfection in both cell lines with (**E**, **G**) a premiR negative control and a premiR-92a-3p or with (**F**, **H**) a LNA negative control and a LNA-miR-92a-3p. **I**–**L** Immunfluorescence showing the effect of miR-92a-3p (**I**, **K**) overexpression or (**J**, **L**) inhibition on Ki-67 expression. Quantitative data were obtained by measuring co-localization of DAPI staining with Ki-67 positive areas using ImageJ software. Data are presented as the mean +/− SEM. *n* = 3 independent experiments. Representative Western blots are shown along with their quantification. *p < 0.05; **p < 0.01; ***p < 0.001. CTL control, LNA Locked Nucleic Acid.

To evaluate the importance of targeting miR-92a-3p in LUAD, we performed additional experiments using two other LUAD cell lines exhibiting distinct mutational profiles (PC9 and H1975, which harbor TP53 and EGFR mutations). Figure 3A–D showed that similarly, inhibition of miR-92a-3p increased BIM expression and the cleavage of caspase 3 following cisplatin treatment. Moreover, transfection with miR-92a-3p mimic increased cell growth and Ki-67 positive cells whereas transfection with miR-92a-3p inhibitor decreased cell proliferation in both cell lines (Fig. 3E–L). Altogether, these results suggest that miR-92a-3p influences LUAD cell sensitivity to cisplatin and proliferation independently of their mutational status.

### Concomitant altered pulmonary expression of both BIM and miR-92a-3p in the KRASG12D-mediated lung cancer mouse model

**Fig. 4:**
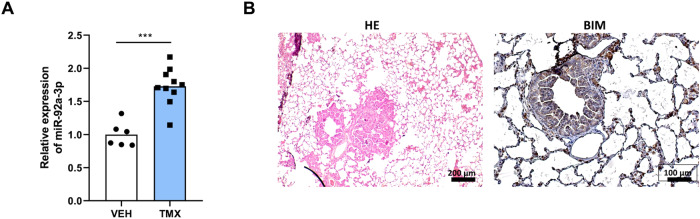
miR-92a-3p and its target BIM are dysregulated in lung tumors of the experimental KRASG12D-mediated lung cancer mouse model. **A** Relative expression of miR-92a-3p in lung tissues of mice treated with tamoxifen (*n* = 10) or vehicle (*n* = 6). **B** Representative images of lung tissue sections assessed by hematoxylin and eosin staining and immunohistochemical analysis of BIM. ***p < 0.001). VEH vehicle, TMX tamoxifen, HE hematoxylin and eosin.

To further strengthen our in vitro findings, expression levels of miR-92a-3p and BIM were assessed in the KRASG12D-mediated lung adenocarcinoma mouse model, which closely resembles the genetic and pathophysiological features of human lung adenocarcinoma. Compared with control, these analyses showed an increased expression of miR-92a-3p in tumor tissue, 15 weeks after tumor induction, along with a reduced tumoral BIM protein expression compared to adjacent non-tumor tissue (Fig. 4).

### miR-92a-3p also influences response of pancreatic cancer cells to cisplatin

**Fig. 5:**
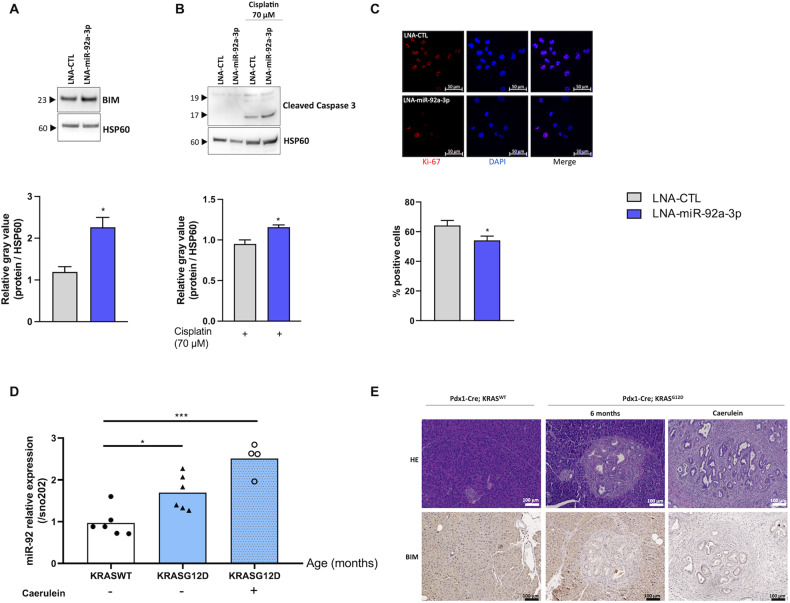
miR-92a-3p influences response of pancreatic cancer cells to cisplatin. **A**, **B** Western blot showing the effect of miR-92a-3p inhibition in PANC1 cells on (**A**) BIM expression and (**B**) the caspase 3 apoptotic response induced by cisplatin (70 µM for 24 h). Representative Western blots are shown along with their quantification. **C** Immunofluorescence showing the effect of miR-92a-3p inhibition on Ki-67 expression. Quantitative data were obtained by measuring co-localization of DAPI staining with Ki-67 areas using ImageJ software. Data are presented as the mean +/− SEM. *n* = 3 independent experiments. **D** Relative expression of miR-92a-3p in tumoral tissues from the KRASG12D-mediated pancreatic cancer mouse model with or without caerulein injections and compared with corresponding wild-type mice. **E** Representative images of mouse pancreatic sections stained with hematoxylin and eosin and immunohistochemical analysis of BIM. Data are presented as the mean +/− SEM. *n* = at least four mice per group. *p < 0.05, ***p < 0.001. WT wild type.

As mutation of *KRAS* is the most frequent genetic alteration in pancreatic adenocarcinoma as well as in precancerous lesions such as PanIN (Pancreatic Intraepithelial neoplasia) [28], we evaluated whether miR-92a-3p also influences response of KRAS-mutated pancreatic cancer cells to cisplatin by performing loss of function experiments in PANC1 cells, a human epithelioid carcinoma cell line harboring the KRASG12D mutation. This showed that downregulation of miR-92a-3p led to the induction of BIM expression and to the increase of caspase 3 cleavage after cisplatin exposure (Fig. 5A, B). Moreover, silencing of miR-92a-3p decreased PANC1 cell proliferation as shown in Fig. 5C.

Expression of miR-92a-3p was also assessed in tumoral tissues from the KRASG12D-mediated pancreatic cancer mouse model and compared with corresponding wild-type animals (Fig. 5D). Figure 5E indicates that mice exhibiting PanIN lesions or invasive pancreatic ductal adenocarcinoma show an increased miR-92a-3p expression and a downregulation of BIM expression.

### Targeting the polycistronic lncRNA MIR17HG has no effect on the sensitivity of LUAD cells to cisplatin

As miR-92a-3p belongs to the polycistronic miRNA cluster miR-17 ~ 92 encoded by MIR17HG, which gives rise to six oncogenic miRNAs grouped into four different families (miR-17, miR-18, miR-19, and miR-92) based on their seed regions [29], we reasoned that directly targeting nuclear MIR17HG would provide a much more efficient approach to promote cisplatin sensitivity by simultaneously inducing the downregulation of all members of the miR-17 ~ 92 cluster. Interestingly, this strategy was recently successfully applied in multiple myeloma using GapmeRs, which consist of single-stranded antisense oligonucleotides able to induce RNase-H cleavage of the targeted transcript especially in the nucleus [30]. We first confirmed the nuclear localization of MIR17HG by RNA-Fish® in A549 cells and its increased expression in the LUAD mouse model (Fig. S2A–B).

We then designed ten distinct GapmeRs directed against human MIR17HG and tested their efficacy in vitro using A549 and PC9 cell lines. As shown in Fig. S2C, among the ten tested GapmeRs, eight were able to efficiently repress nuclear MIR17HG 48 h after transfection. Nevertheless, two of them exhibited strong cellular toxicity likely due to off-target effects and were thus excluded. We then assessed whether the six selected GapmeRs significantly alter the expression level of the miRNAs including in the miR-17 ~ 92 cluster. As depicted in Fig. S2C, each of the six selected GapmeRs reproducibly but modestly induced the downregulation of all members of the cluster 72 h after transfection in A549 cells (Fig. S2C) and in PC9 cells (data not shown). However, in contrast to the study from Morelli et al. [31], none of the selected GapmeRs was able to affect BIM expression or cisplatin sensitivity (Fig. S2D).

## Discussion

Cancer therapy usually relies on the simultaneous or sequential administration of chemotherapeutic agents with non-overlapping mechanisms of action to achieve greater treatment efficacy and to prevent the selection of drug resistant clones as well as the subsequent early regrowth of tumors [32, 33]. Nevertheless, despite remarkable successes in some forms of lymphoma, breast and testicular cancers, most patients receiving polychemotherapy inevitably relapse [34]. Therefore, new insights into the molecular determinants of tumor sensitivity to chemotherapy are critical to optimize current treatment regimens and to develop new effective combination therapies. This is particularly true for mutant KRAS-driven lung tumors, which are usually refractory to first-line platinum-based chemotherapy [35]. In this study, we reasoned that miRNA-based combination therapy represents an attractive therapeutic strategy to increase cisplatin response of KRAS-driven lung cancer. Indeed, the ability of miRNAs to modulate multiple transcripts from the same or interconnected oncogenic pathways represents a steady advantage over traditional single gene approaches [36].

Functional genetic screening is a powerful unbiased strategy for assessing the role of specific genes in different neoplastic phenotypes, and the rapid identification of novel drug targets [37, 38]. Indeed, this approach has the great advantage of enabling the establishment of a causative link between gene expression and a specific oncogenic phenotype such as response to anticancer agents, in contrast to many other profiling high-throughput methods that are mainly correlative [37]. In line with this, we designed a miRNA loss-of-function drug sensitization screen to uncover miRNAs whose loss-of-function silencing reduces the viability of *KRAS* mutated A549 cells in the presence of a sublethal concentration of cisplatin. Among the various miRNAs investigated, we focused our attention on miR-92a-3p, whose decreased expression has the greatest impact on cisplatin sensitivity. Indeed, miR-92a-3p is a well-characterized small oncogenic ncRNA whose pro-tumoral activity has been characterized in a variety of distinct human malignancies [39–41]. In particular, this ncRNA is almost invariably overexpressed in tumors and represents an important regulator of numerous tumorigenic processes including cell proliferation and apoptosis through various molecular mechanisms that can be shared by cancer cells or unique to a given tumor type [42–44]. For example, miR-92a-3p has been shown to inhibit apoptosis and tumor growth in many distinct malignancies by targeting the BH3-only protein BIM and PTEN respectively [23, 27], while promoting cell proliferation by cancer-type specific mechanisms involving targeting of genes such as BTG2 in breast cancer or FBXW7 in renal cancer [39, 45]. In lung cancer, previous reports have notably shown the diagnostic/prognostic value of miR-92a-3p as well as a significant association between expression of this miRNA and response to radiotherapy [46–49]. Nevertheless, although several in vitro evidence support an oncogenic role of miR-92a-3p in lung cancer cells [50], its precise molecular function remains unclear and little is known about the role played by miR-92a-3p in chemoresistance. In this study, we showed that miR-92a-3p, using gain- and loss-of-function approaches, is an important determinant of LUAD sensitivity to cisplatin not only in cells in which *KRAS* is mutated but also in those harboring alteration of *TP53*. This is indeed of particular interest, as *TP53* somatic mutations frequently occur in LUAD patients and are usually associated with resistance to therapy including cisplatin-based treatment regimens [51].

Mechanistically, we showed that miR-92a-3p influences, at least in part, cisplatin sensitivity by fine-tuning the BH3 only protein BIM, a central activator of apoptosis [52]. This is consistent with a previous study that linked loss of expression of BIM with cisplatin resistance in ovarian cancer [52]. Interestingly, we also showed that silencing of miR-92a-3p also increases BIM protein expression and sensitivity to cisplatin in another KRAS-driven tumor cell line model distinct from LUAD, the pancreatic ductal adenocarcinoma. As this malignancy is also usually refractory to therapies, our results warrant additional investigations to evaluate whether the targeting of miR-92a-3p could represent a new therapeutic option, especially as cisplatin is not used as first-line treatment in this disease. Finally, our results also showed that modulation of miR-92a-3p expression not only influences apoptosis but also cancer cell proliferation likely by regulating the PTEN/AKT signaling pathway. Thus, miR-92a-3p is involved in the regulation of distinct biological cell processes essential for the initiation and maintenance of the malignant phenotype [53].

As miR-92a-3p belongs to the polycistronic miRNA cluster miR-17 ~ 92 encoded by *MIR17HG*, we hypothesized that directly targeting MIR17HG using a single GapmeR, would represent a better strategy to promote cisplatin sensitivity by simultaneously inducing the downregulation of the six miRNAs encoded by miR-17 ~ 92. Indeed, it is well-established that the oncogenic activity of the cluster primarily relies on its cognate miRNAs that efficiently modulate multiple oncogenic pathways by regulating a repertoire of targets that can be shared by members of this cluster or specific to an individual miRNA. However, in contrast to Morelli et al. (2018) who recently successfully applied this approach in multiple myeloma, none of the GapmeRs tested was able to modulate BIM expression or promote the sensitivity of LUAD to cisplatin [31]. This may be explained by an addiction of multiple myeloma-derived cells to MIR17HG or an off-target effect of the GapmeR designed by Morelli et al. (2018) [31]. Therefore, additional works should be performed to better understand these discrepancies.

In conclusion, our results support a fundamental role of miR-92a-3p in the regulation of the apoptotic process which may be therapeutically exploited to promote the sensitivity of cancer cells to cisplatin.

## Methods

### Cell culture

Human non-small cell lung cancer (NSCLC) A549 cells (ATCC, LGC Standards S.A.R.L, Molsheim, France) and human pancreatic epithelial carcinoma PANC1 cells (ATCC) were cultured in Glutamax-containing DMEM (Thermofisher Scientific, Illkirch-Graffenstaden, France) medium supplemented with 10% FBS (Thermofisher Scientific) and 1% Penicillin/Streptomycin (Thermofisher Scientific). Human NSCLC PC9 (ATCC) and H1975 (ATCC) cells were cultured in Glutamax-containing RPMI (Thermofisher Scientific) supplemented as above. Cells were cultured at 37 °C in a humidified atmosphere of 5% CO_2_ in T75 flasks and seeded into 6-, 24- or 96-well plates 24 h before the experiments.

### Functional screening

A library of LNA-based miRNA inhibitors (Qiagen, miRbase version 10, Les Ulis, France) against the most highly expressed miRNAs in A549 cells was used in this study (Supplemental Table 1), which were identified by small RNA seq on SOLID 5500 WF (Thermofisher Scientific). Each LNA inhibitor (10 nM) was transfected in triplicate in A549 cells using Lipofectamine RNAiMAX (Thermofisher Scientific) in 96-well plates. After 48 hours, cells were exposed to a sub-apoptotic dose of cisplatin (30 μM for three days). Viability was assessed using the CellTiterGlo assay (Promega, Charbonnières-les-Bains, France). Normalization was carried out by dividing each sample value by the median of all samples on the plate (the majority of sample wells will thus serve as a reference). miRNA candidates were selected for further analysis based on statistical significance (*p* < 0.01) and degree of sensibility induced (assessed by viability after drug exposure, normalized viability below 3).

### Small RNA-Seq

Small RNA-Seq was performed as described in [54]. Briefly, 500 ng of total RNAs were ligated, reverse transcribed and amplified (18 cycles) with the reagents from the NextFlex small RNAseq kit V3 (Bioo scientific, Villebon-sur-Yvette, France). Amplified libraries were quantified with the Bioanalyzer High Sensitivity DNA Kit (Agilent, Les Ulis, France), pooled and size-selected from 140 nt to 170 nt with the LabChip XT DNA 300 Assay Kit (Caliper Lifesciences, Villepinte, France). Libraries were then sequenced on an Illumina Nextseq 500 Mid Flowcell with 75 pb reads for a total of 147 M reads.

### Transfection and cisplatin exposure

Cells were transfected at 30 to 40% of confluency in 6-, 24- or 96-well plates, using Lipofectamine RNAiMAX (Thermofisher Scientific) with pre-miRNAs and control miRNA (Thermofisher Scientific) or LNA inhibitors and LNA negative control (Qiagen) at a final concentration of 10 nM. Antisens oligonucleotides GapmeRs, directed against MIR17HG (Supplementary Table 2; Qiagen) and siRNA directed against BIM or PTEN (ThermoFisher Scientific) were transfected as described above at a final concentration of 25 nM and 10 nM respectively.

Forty-eight hours after transfection, cells were exposed to a sub-apoptotic dose of cisplatin at 30 μM or 70 µM for 24 h for NSCLC cell lines and PANC1 respectively.

### Protein extraction and immunoblotting

Cells were lysed in RIPA (Radioimmunoprecipitation assay) buffer (Sigma-Aldrich, Saint-Quentin-Fallavier, France) containing protease and phosphatase inhibitors (Roche, Meylan, France). Lysates were quantified using the BCA (Bicinchoninic acid) protein assay kit (Thermofisher Scientific). Proteins were separated by SDS-polyacrylamide gel and transferred onto nitrocellulose membranes (Biorad, Marnes-la-Coquette, France). The membranes were blocked with 5% fat free milk or Bovine Serum Albumin (BSA, in case of phospho protein) in Tris-buffered Saline (TBS) containing 0.1% Tween-20 (TBS-T) and subsequently incubated with their respective primary antibodies overnight at 4 °C. After washing with TBS-T, membranes were further incubated with horseradish peroxidase-conjugated secondary antibodies for 45 min, followed by washing with TBS-T. Protein bands were visualized with Amersham ECL substrates (GE Healthcare, Buc, France).

The following antibodies were used: goat anti-HSP60 (sc-1052, Santa Cruz Biotechnology Inc., Heidelberg, Germany), rabbit anti-cleaved Caspase 3 (Asp175) (#9661, Cell Signaling Technology, Saint-Cyr-L’École, France), rabbit anti-Bim (#2933, Cell Signaling Technology), rabbit anti-PTEN (#9559, Cell Signaling Technology), rabbit anti-pAKT (#4058, Cell Signaling Technology), rabbit anti-AKT (#9272, Cell Signaling Technology).

### Immunofluorescence analysis

Cells were grown on a Round Glass Coverslip Ø 16 mm (Thermofisher Scientific) placed inside a 24-well plate. Coverslips were washed in phosphate-buffered saline (PBS) and fixed in 4% paraformaldehyde for 15 min, then cells were permeabilized using 0.1% Triton X-100 (Agilent Technologies, Les Ulis, France) for 10 min and blocked with 3% BSA in TBS for 30 min. Incubation with primary antibody rabbit anti-Ki-67 (#9129, Cell Signaling Technology) was performed in a blocking solution BSA (1%) at 37 °C for 1 h. After three washes with TBS, cells were incubated with secondary antibodies for 45 min at 37 °C. Coverslips were then fixed on microscope slides using ProLong Gold Antifade Reagent with DAPI (Thermofisher Scientific). Fluorescence was detected with a Spinning Disk confocal microscope (Zeiss, Rueil Malmaison, France). Quantitative data were obtained by measuring co-localization of DAPI staining with Ki-67 areas using ImageJ software (National Institute of Health).

### Cell proliferation

Cells were grown in 6-well plates and then transfected. After 24 h, cells were seeded into 96-well plates. Cell proliferation was evaluated after three days using the CellTiterGlo kit (Promega).

### Clonogenicity

Cells were grown in 6-well plates and then transfected. After 24 h, 4000 cells were seeded into 6-well plates for four and seven days. Cells were fixed with cold methanol and stained with 0.5% crystal violet (Sigma-Aldrich). Excess stain was removed by washing repeatedly with water. For colony quantification, the crystal violet staining was solubilized using 10% acetic acid and the absorbance was measured at a wavelength of 590 nm.

### RNA Fish

Custom Stellaris® FISH Probes were designed against MIR17HG using the Stellaris® RNA FISH Probe Designer (Biosearch Technologies, Hoddesdon, United-Kingdom) available online at https://www.biosearchtech.com.

A549 cells were grown in μ-Dish 35 mm glass bottom (Ibidi, Nanterre, France). After 48 h of culture, cells were permeabilized using 70% ethanol and then hybridized with the MIR17HG Stellaris RNA FISH Probe set labeled with CAL Fluor Red 590 (Biosearch Technologies), following the manufacturer’s instructions at the final concentration of 500 nM. Acquisition was performed using an inverted confocal microscope LSM 710 (Zeiss) with the high-resolution module AiryScan (Zeiss) and a 63x/1.4 oil immersion lens. Images were processed using Zen software (Zeiss).

### Mouse models of cancer

All animal care and experimental protocols were approved by the Institutional Animal Care and Use Committee (IACUC) of Lille University (Protocol Number: APAFIS#17098.05 and APAFIS#00422.02). Sample size was chosen empirically based on our previous experiences in the calculation of experimental variability; no statistical method was used to predetermine sample size and no samples, mice or data points were excluded from the reported analyses. Experiments were performed non-blinded using treatment group randomized mice. Manipulators carried out all experimental protocols under strict guidelines to ensure careful and consistent handling of the mice.

#### CCSP-Cre; LSL-KRAS^G12D^ lung adenocarcinoma mouse model

CCSP-Cre and LSL-KRAS^G12D^ mice were purchased from Jackson laboratory (L’Arbresle, France). To induce lung tumors, tamoxifen (0.25 mg/g) (Sigma Aldrich) was injected in CCSP-Cre; LSL-KRAS^G12D^ mice (8 to 12 weeks old males) for 5 days. Fifteen weeks after tumor initiation, mice were sacrificed by cervical dislocation. For histological analysis, lungs were perfused with 10% formalin (Sigma-Aldrich) and then included in paraffin. For molecular biology analysis, lungs were stored in RNAlater solution (Thermofisher Scientific).

#### Pdx1-Cre; LSL-KRASG12D pancreatic cancer mouse model

Pdx1-Cre; LSL-KRASG12D mice were a generous gift from Dr Nicolas Jonckheere (UMR9020 CNRS – U1277 Inserm).

To induce pancreatic tumors, intraperitoneal injections of 37.5 µg/mL caerulein (Sigma-Aldrich) solution were performed using 6-month-old transgenic mice following a two-step protocol. First, an acute treatment consisting of an injection every hour for 6 h (1st day) followed by a chronic treatment consisting of caerulein injections 5 days a week for 59 days. After sacrifice, pancreas were collected from 6-month-old KRASG12D males and WT control mice, fixed and embedded in paraffin for histological analysis or flash-frozen for RNA extraction.

### RNA extraction

Total RNAs were extracted from tissue or cell samples with the miRNeasy Mini kit (Qiagen), according to the supplier’s recommendations.

### Quantitative RT-PCR

For cell samples, miRNA retro-transcription was performed using RT miRCURY LNA kit (Qiagen). Quantitative PCR was performed on a StepOnePlus^TM^ Real-Time PCR System (Thermofisher Scientific) with miRCURY LNA Probe PCR kit (Qiagen) and with the following primers: miR-17-5p (YP02119304), miR-18a-5p (YP00204207), miR-19a-3p (YP00205862), miR-19b-3p (YP00204450), miR-20a-5p (YP00204292), miR-92a-3p (YP00204258). For normalization, transcript levels of RNU44 (YP00203902) were used as endogenous control for miRNA expression. For mouse samples, miRNA retro-transcription was performed using the TaqMan™ MicroRNA Reverse Transcription Kit (Thermofisher Scientific). Quantitative PCR was performed on a StepOnePlus^TM^ Real-Time PCR System using Universal Master Mix (Thermofisher Scientific) and the following TaqMan assays: miR-92a-3p (assay ID 000430), snoRNA202 (assay ID 001232) and snoRNA251 (assay ID 001236).

mRNA retro-transcription was performed using the High Capacity cDNA reverse transcription kit (Thermofisher Scientific). Expression levels of genes (primers are listed in Supplementary Table 3) were performed on a StepOnePlus^TM^ Real-Time PCR System (Thermofisher Scientific) with Fast SYBR Green Master Mix (Thermofisher Scientific). For normalization, transcript levels of PPIA were used as endogenous control for gene expression.

Relative expression levels of mRNAs and miRNAs were assessed using the comparative threshold cycle method (2^−ΔΔCT^) [55].

### Histology

5-μm paraffin-embedded sections were mounted and stained with hematoxylin and eosin (Sigma-Aldrich). Acquisition was performed using a DMi8 microscope (Leica, Nanterre, France) at 20x magnification.

### Immunohistochemistry

5-μm paraffin-embedded sections were sequentially incubated in xylene (5 min twice), 100% ethyl alcohol (5 min twice), 95% ethyl alcohol (5 min twice), and 80% ethyl alcohol (5 min). After washing with water, the sections were antigen-retrieved using citrate buffer (pH 6.0; DAKO, Les Ulis, France) in a domestic microwave oven for 20 min and cooled to ambient temperature. Sections were then washed with TBS-T and quenched with 3% hydrogen peroxide in TBS for 10 min, blocked for avidin/biotin activity, blocked with serum-free blocking reagent, and incubated with primary antibody rabbit anti BIM (#C34C5, Cell Signaling Technology). Immunohistochemical staining was developed using the DAB (3,3’-Diaminobenzidine) substrate system (DAKO). Acquisition was performed using a DMi8 microscope (Leica) at 20x magnification.

### Statistical analysis

Statistical analyses were performed using GraphPad Prism software. Results are given as mean ± SEM. Two-tailed Mann-Whitney test was used for single comparisons; one-way ANOVA followed by Bonferroni post hoc test was used for multiple comparisons. *p*-value less than 0.05 was considered statistically significant.

## Supplementary information

Supplemental Tables and Figures

Uncropped WB

Reproducibility checklist

Autorship change approval

## Data Availability

All data generated or analyzed during this study are available from the corresponding author on reasonable request.

## References

[1] Zappa C, Mousa SA (2016). Non-small cell lung cancer: current treatment and future advances. Transl Lung Cancer Res.

[2] Schulze AB, Evers G, Kerkhoff A, Mohr M, Schliemann C, Berdel WE (2019). Future options of molecular-targeted therapy in small cell lung cancer. Cancers..

[3] Dutt A, Wong KK (2006). Mouse models of lung cancer. Clin Cancer Res Off J Am Assoc Cancer Res.

[4] Ocak S, Sos ML, Thomas RK, Massion PP (2009). High-throughput molecular analysis in lung cancer: insights into biology and potential clinical applications. Eur Respir J.

[5] Kwon MC, Berns A (2013). Mouse models for lung cancer. Mol Oncol.

[6] Pikor LA, Ramnarine VR, Lam S, Lam WL (2013). Genetic alterations defining NSCLC subtypes and their therapeutic implications. Lung Cancer Amst Neth.

[7] Shim HS, Choi YL, Kim L, Chang S, Kim WS, Roh MS (2017). Molecular testing of lung cancers. J Pathol Transl Med.

[8] Herbst RS, Morgensztern D, Boshoff C (2018). The biology and management of non-small cell lung cancer. Nature..

[9] Imyanitov EN, Iyevleva AG, Levchenko EV (2021). Molecular testing and targeted therapy for non-small cell lung cancer: current status and perspectives. Crit Rev Oncol Hematol.

[10] Hirsch FR, Scagliotti GV, Mulshine JL, Kwon R, Curran WJ, Wu YL (2017). Lung cancer: current therapies and new targeted treatments. Lancet Lond Engl.

[11] Lemjabbar-Alaoui H, Hassan OU, Yang YW, Buchanan P (2015). Lung cancer: Biology and treatment options. Biochim Biophys Acta.

[12] Kato S, Fujiwara Y, Hong DS (2022). Targeting KRAS: crossroads of signaling and immune inhibition. J Immunother Precis Oncol.

[13] Seguin L, Durandy M, Feral CC (2022). Lung adenocarcinoma tumor origin: a guide for personalized medicine. Cancers..

[14] Amable L (2016). Cisplatin resistance and opportunities for precision medicine. Pharmacol Res.

[15] Lin JJ, Shaw AT (2016). Resisting resistance: targeted therapies in lung cancer. Trends Cancer.

[16] Galluzzi L, Vitale I, Michels J, Brenner C, Szabadkai G, Harel-Bellan A (2014). Systems biology of cisplatin resistance: past, present and future. Cell Death Dis.

[17] Liu WJ, Du Y, Wen R, Yang M, Xu J (2020). Drug resistance to targeted therapeutic strategies in non-small cell lung cancer. Pharmacol Ther.

[18] Landau DA, Slack FJ (2011). MicroRNAs in mutagenesis, genomic instability, and DNA repair. Semin Oncol.

[19] Peng Y, Croce CM (2016). The role of MicroRNAs in human cancer. Signal Transduct Target Ther.

[20] Reda El Sayed S, Cristante J, Guyon L, Denis J, Chabre O, Cherradi N (2021). MicroRNA therapeutics in cancer: current advances and challenges. Cancers..

[21] Christopher AF, Kaur RP, Kaur G, Kaur A, Gupta V, Bansal P (2016). MicroRNA therapeutics: discovering novel targets and developing specific therapy. Perspect Clin Res.

[22] Leung ELH, Luo LX, Liu ZQ, Wong VKW, Lu LL, Xie Y (2018). Inhibition of KRAS-dependent lung cancer cell growth by deltarasin: blockage of autophagy increases its cytotoxicity. Cell Death Dis.

[23] Niu H, Wang K, Zhang A, Yang S, Song Z, Wang W (2012). miR-92a is a critical regulator of the apoptosis pathway in glioblastoma with inverse expression of BCL2L11. Oncol Rep.

[24] Sionov RV, Vlahopoulos SA, Granot Z (2015). Regulation of Bim in health and disease. Oncotarget..

[25] Crowley LC, Waterhouse NJ. Detecting cleaved caspase-3 in apoptotic cells by flow cytometry. Cold Spring Harb Protoc. 2016;2016. 10.1101/pdb.prot087312 27803251

[26] Yu JSL, Cui W (2016). Proliferation, survival and metabolism: the role of PI3K/AKT/mTOR signalling in pluripotency and cell fate determination. Development..

[27] Lu C, Shan Z, Hong J, Yang L (2017). MicroRNA-92a promotes epithelial-mesenchymal transition through activation of PTEN/PI3K/AKT signaling pathway in non-small cell lung cancer metastasis. Int J Oncol.

[28] Waters AM, Der CJ (2018). KRAS: the critical driver and therapeutic target for pancreatic cancer. Cold Spring Harb Perspect Med.

[29] Ota A, Tagawa H, Karnan S, Tsuzuki S, Karpas A, Kira S (2004). Identification and characterization of a novel gene, C13orf25, as a target for 13q31-q32 amplification in malignant lymphoma. Cancer Res.

[30] Amodio N, Stamato MA, Juli G, Morelli E, Fulciniti M, Manzoni M (2018). Drugging the lncRNA MALAT1 via LNA gapmeR ASO inhibits gene expression of proteasome subunits and triggers anti-multiple myeloma activity. Leukemia..

[31] Morelli E, Biamonte L, Federico C, Amodio N, Di Martino MT, Gallo (2018). Therapeutic vulnerability of multiple myeloma to MIR17PTi, a first-in-class inhibitor of pri-miR-17-92. Blood..

[32] Pritchard JR, Lauffenburger DA, Hemann MT (2012). Understanding resistance to combination chemotherapy. Drug Resist Updat Rev Comment Antimicrob Anticancer Chemother.

[33] Yap TA, Parkes EE, Peng W, Moyers JT, Curran MA, Tawbi HA (2021). Development of immunotherapy combination strategies in cancer. Cancer Discov.

[34] Chatterjee N, Bivona TG (2019). Polytherapy and Targeted Cancer Drug Resistance. Trends Cancer.

[35] Xie M, Xu X, Fan Y (2021). KRAS-Mutant Non-Small Cell Lung Cancer: An Emerging Promisingly Treatable Subgroup. Front Oncol.

[36] Garzon R, Marcucci G, Croce CM (2010). Targeting microRNAs in cancer: rationale, strategies and challenges. Nat Rev Drug Discov.

[37] Iorns E, Lord CJ, Turner N, Ashworth A (2007). Utilizing RNA interference to enhance cancer drug discovery. Nat Rev Drug Discov.

[38] Haley B, Roudnicky F (2020). Functional genomics for cancer drug target discovery. Cancer Cell.

[39] Jinghua H, Qinghua Z, Chenchen C, Lili C, Xiao X, Yunfei W (2021). MicroRNA miR-92a-3p regulates breast cancer cell proliferation and metastasis via regulating B-cell translocation gene 2 (BTG2). Bioengineered..

[40] Wang Y, Chen A, Zheng C, Zhao L (2021). miR-92a promotes cervical cancer cell proliferation, invasion, and migration by directly targeting PIK3R1. J Clin Lab Anal.

[41] Yang J, Hai J, Dong X, Zhang M, Duan S (2021). MicroRNA-92a-3p enhances cisplatin resistance by regulating Krüppel-Like factor 4-mediated cell apoptosis and epithelial-to-mesenchymal transition in cervical cancer. Front Pharmacol.

[42] Olive V, Jiang I, He L (2010). mir-17-92, a cluster of miRNAs in the midst of the cancer network. Int J Biochem Cell Biol.

[43] Zhang X, Li Y, Qi P, Ma Z (2018). Biology of MiR-17-92 cluster and its progress in lung cancer. Int J Med Sci.

[44] Zhao W, Gupta A, Krawczyk J, Gupta S (2022). The miR-17-92 cluster: Yin and Yang in human cancers. Cancer Treat Res Commun.

[45] Zeng R, Huang J, Sun Y, Luo J (2020). Cell proliferation is induced in renal cell carcinoma through miR-92a-3p upregulation by targeting FBXW7. Oncol Lett.

[46] Huang YF, Liu MW, Xia HB, He R (2022). Expression of miR-92a is associated with the prognosis in non-small cell lung cancer: an observation study. Medicine (Baltimore).

[47] Reis PP, Drigo SA, Carvalho RF, Lopez Lapa RM, Felix TF, Patel D (2020). Circulating miR-16-5p, miR-92a-3p, and miR-451a in plasma from lung cancer patients: potential application in early detection and a regulatory role in tumorigenesis pathways. Cancers..

[48] Vykoukal J, Fahrmann JF, Patel N, Shimizu M, Ostrin EJ, Dennison JB (2022). Contributions of circulating microRNAs for early detection of lung cancer. Cancers..

[49] Zeng L, Zeng G, Ye Z (2022). Bioinformatics analysis for identifying differentially expressed MicroRNAs derived from plasma exosomes associated with radiotherapy resistance in non-small-cell lung cancer. Appl Bionics Biomech.

[50] Alcantara KMM, Garcia RL (2019). MicroRNA‑92a promotes cell proliferation, migration and survival by directly targeting the tumor suppressor gene NF2 in colorectal and lung cancer cells. Oncol Rep.

[51] Mogi A, Kuwano H (2011). TP53 mutations in nonsmall cell lung cancer. BioMed Res Int.

[52] Wang J, Zhou JY, Wu GS (2011). Bim protein degradation contributes to cisplatin resistance. J Biol Chem.

[53] Taieb J, Prager GW, Melisi D, Westphalen CB, D’Esquermes N, Ferreras A (2020). First-line and second-line treatment of patients with metastatic pancreatic adenocarcinoma in routine clinical practice across Europe: a retrospective, observational chart review study. ESMO Open.

[54] Savary G, Dewaeles E, Diazzi S, Buscot M, Nottet N, Fassy J (2019). The long noncoding RNA DNM3OS is a reservoir of FibromiRs with major functions in lung fibroblast response to TGF-β and pulmonary fibrosis. Am J Respir Crit Care Med.

[55] Livak KJ, Schmittgen TD (2001). Analysis of relative gene expression data using real-time quantitative PCR and the 2(-Delta Delta C(T)) Method. Methods San Diego Calif.

