# Supplementary material for: miR-92a-3p regulates cisplatin-induced cancer cell death
Source: Cell Death Dis. 2023 Sep 13;14(9):603. doi: 10.1038/s41419-023-06125-z (PMC10499794; doi:10.1038/s41419-023-06125-z)
Supplement: Supplementary file 1 — Supplemental Tables and Figures [file 41419_2023_6125_MOESM1_ESM.docx]

**Supplemental Table 1. List of 138 LNA-based miRNA inhibitors against the most expressed miRNAs in A549 cells.**

|  | **miRNA** |  |  | **miRNA** |
| --- | --- | --- | --- | --- |
| 1 | hsa-miR-151-3p |  | 73 | hsa-miR-22 |
| 2 | hsa-miR-16-2 |  | 74 | hsa-miR-19a |
| 3 | hsa-miR-193b |  | 75 | hsa-miR-18a |
| 4 | hsa-miR-21 |  | 76 | hsa-miR-29a |
| 5 | hsa-miR-24-2 |  | 77 | hsa-miR-27b |
| 6 | hsa-miR-28-3p |  | 78 | hsa-miR-19b |
| 7 | hsa-miR-345 |  | 79 | hsa-miR-200b |
| 8 | hsa-miR-34a |  | 80 | hsa-miR-196a |
| 9 | hsa-miR-34b |  | 81 | hsa-miR-23b |
| 10 | hsa-miR-374a |  | 82 | hsa-miR-424 |
| 11 | hsa-miR-424 |  | 83 | hsa-miR-34a |
| 12 | hsa-miR-452 |  | 84 | hsa-miR-374b |
| 13 | hsa-miR-452 |  | 85 | hsa-miR-30e |
| 14 | hsa-miR-454 |  | 86 | hsa-miR-532-5p |
| 15 | hsa-miR-455-3p |  | 87 | hsa-miR-484 |
| 16 | hsa-miR-500a |  | 88 | hsa-miR-340 |
| 17 | hsa-miR-550a |  | 89 | hsa-miR-31 |
| 18 | hsa-miR-597 |  | 90 | hsa-miR-331-3p |
| 19 | hsa-miR-660 |  | 91 | hsa-miR-423-3p |
| 20 | hsa-miR-935 |  | 92 | hsa-miR-30c |
| 21 | hsa-miR-1180 |  | 93 | hsa-miR-30d |
| 22 | hsa-let-7i |  | 94 | hsa-miR-324-5p |
| 23 | hsa-let-7c |  | 95 | hsa-miR-423-5p |
| 24 | hsa-let-7b |  | 96 | hsa-miR-361-5p |
| 25 | hsa-miR-151-5p |  | 97 | hsa-miR-450a |
| 26 | hsa-let-7a |  | 98 | hsa-miR-30e |
| 27 | hsa-miR-10a |  | 99 | hsa-miR-335 |
| 28 | hsa-miR-148b |  | 100 | hsa-miR-425 |
| 29 | hsa-miR-143 |  | 101 | hsa-miR-339-5p |
| 30 | hsa-miR-15a |  | 102 | hsa-miR-320a |
| 31 | hsa-miR-181b |  | 103 | hsa-miR-320a |
| 32 | hsa-let-7a |  | 104 | hsa-miR-378 |
| 33 | hsa-let-7d |  | 105 | hsa-miR-7 |
| 34 | hsa-let-7f |  | 106 | hsa-miR-96 |
| 35 | hsa-let-7i |  | 107 | hsa-miR-9 |
| 36 | hsa-miR-125a-5p |  | 108 | hsa-miR-582-5p |
| 37 | hsa-miR-15b |  | 109 | hsa-miR-99a |
| 38 | hsa-miR-181a |  | 110 | hsa-miR-93 |
| 39 | hsa-miR-128 |  | 111 | hsa-miR-99b |
| 40 | hsa-miR-125b |  | 112 | hsa-miR-671-5p |
| 41 | hsa-miR-126 |  | 113 | hsa-miR-103-2 |
| 42 | hsa-miR-100 |  | 114 | hsa-miR-7-1 |
| 43 | hsa-miR-101 |  | 115 | hsa-miR-92a-3p |
| 44 | hsa-miR-140-3p |  | 116 | hsa-miR-652 |
| 45 | hsa-miR-106b |  | 117 | hsa-miR-23c |
| 46 | hsa-miR-15b |  | 118 | hsa-miR-98 |
| 47 | hsa-miR-138 |  | 119 | hsa-miR-17 |
| 48 | hsa-miR-16 |  | 120 | hsa-miR-1260 |
| 49 | hsa-miR-146b-5p |  | 121 | hsa-miR-103 |
| 50 | hsa-let-7g |  | 122 | hsa-miR-320b |
| 51 | hsa-miR-106b |  | 123 | hsa-miR-224 |
| 52 | hsa-miR-25 |  | 124 | hsa-miR-449c |
| 53 | hsa-miR-27a |  | 125 | hsa-miR-500b |
| 54 | hsa-miR-185 |  | 126 | hsa-miR-3182 |
| 55 | hsa-miR-224 |  | 127 | hsa-miR-29c |
| 56 | hsa-miR-27b |  | 128 | hsa-miR-365 |
| 57 | hsa-miR-28-5p |  | 129 | hsa-miR-92b |
| 58 | hsa-miR-26b |  | 130 | hsa-miR-339-3p |
| 59 | hsa-miR-194 |  | 131 | hsa-let-7e |
| 60 | hsa-miR-210 |  | 132 | hsa-miR-342-3p |
| 61 | hsa-miR-26a |  | 133 | hsa-miR-140-5p |
| 62 | hsa-miR-191 |  | 134 | hsa-miR-24 |
| 63 | hsa-miR-200a |  | 135 | hsa-miR-590-3p |
| 64 | hsa-miR-30a |  | 136 | hsa-miR-192 |
| 65 | hsa-miR-29b |  | 137 | hsa-miR-31 |
| 66 | hsa-miR-22 |  | 138 | hsa-miR-30b |
| 67 | hsa-miR-182 |  |  |  |
| 68 | hsa-miR-20a |  |  |  |
| 69 | hsa-miR-222 |  |  |  |
| 70 | hsa-miR-221 |  |  |  |
| 71 | hsa-miR-21 |  |  |  |
| 72 | hsa-miR-183 |  |  |  |

**Supplemental Table 2. ID and sequences of GapmeRs**

| **GapmeR ID** | **Sequences** |
| --- | --- |
| LG00197374-DDA (GapmeR 1) | 3’-AGATACACAGTTAGGT-5’ |
| LG00197375-DDA (GapmeR 2) | 3’-TGTGATCCAATCTGGT-5’ |
| LG00197376-DDA (GapmeR 3) | 3’-GTCAAAGTGCTTACAG-5’ |
| LG00197377-DDA (GapmeR 4) | 3’-AGGACAGTATGTGCAC-5’ |
| G00197378-DDA (GapmeR 5) | 3’-TTATTGTGTCGATGTA-5’ |
| LG00197379-DDA (GapmeR 6) | 3’-ATGACTTCTAAAGCTG-5’ |
| LG00197380-DDA (GapmeR 7) | 3’-GTGTGACTGAGGACTG-5’ |
| LG00197381-DDA (GapmeR 8) | 3’-CTTAAAAAGTTCCGGC-5’ |
| LG00197382-DDA (GapmeR 9) | 3’-GACTAAATTGCCTTTA-5’ |
| LG00197383-DDA (GapmeR 10) | 3’-GTTTTTGTCTCTAGCC-5’ |

**Supplemental Table 3. Sequences of primers**

| **Primer** | **Sequences** |
| --- | --- |
| MIR17HG (human) | sens : CTGCTTGCAAAGTGTTGGTG  antisens : AGGCTGCATTTTGTCAGGAG |
| PPIA (human) | sens : GACCCAACACAAATGGTTCC  antisens : GGCCTCCACAATATTCATGC |
| MIR17HG (mouse) | sens : AAACAGCTCAGTTGGGCAAG  antisens : GTGCACAGAGCAAAGCAATC |
| PPIA (mouse) | sens : GACCAAACACAAACGGTTCC  antisens : TTCACCTTCCCAAAGACCAC |

Fig S1

**Figure S1. Silencing of miR-92a-3p in BIM-depleted cells promotes cisplatin-induced apoptosis.** Western blot showing the effect of cisplatin-induced apoptosis assessed by cleaved caspase 3 expression following inhibition of miR-92a-3p in BIM depleted-A549 cells. Data are presented as the mean +/- SEM. n = 3 independent experiments. * : p<0,05 ; *** : p<0,001. CTL : control ; LNA : Locked Nucleic Acid.

Fig S2

**Figure S2. Targeting MIR17HG has no effect on the cell sensitivity to cisplatin.** (A) RNA Fish (Stellaris®) showing the nuclear localization of MIR17HG in cells. (B) Relative expression of MIR17HG and the six miRNAs of the cluster in cells transfected 72h with GapmeRs. (C) Relative expression of MIR17HG in lung tissues of mice with tamoxifen (n = 10) compared to mice with vehicle (n = 6). (D) Western blots showing the effect of MIR17HG inhibition on BIM expression and on the caspase 3 apoptotic response induced by cisplatin (30 µM for 24h). n = 3 independent experiments; * : p<0,05 ; ** : p<0,01. Gp : GapmeR ; CTL : control.
