# Supplementary material for: miR-92a-3p regulates cisplatin-induced cancer cell death
Source: Cell Death Dis. 2023 Sep 13;14(9):603. doi: 10.1038/s41419-023-06125-z (PMC10499794; doi:10.1038/s41419-023-06125-z)

Uncropped Western Blots

Figure 2A

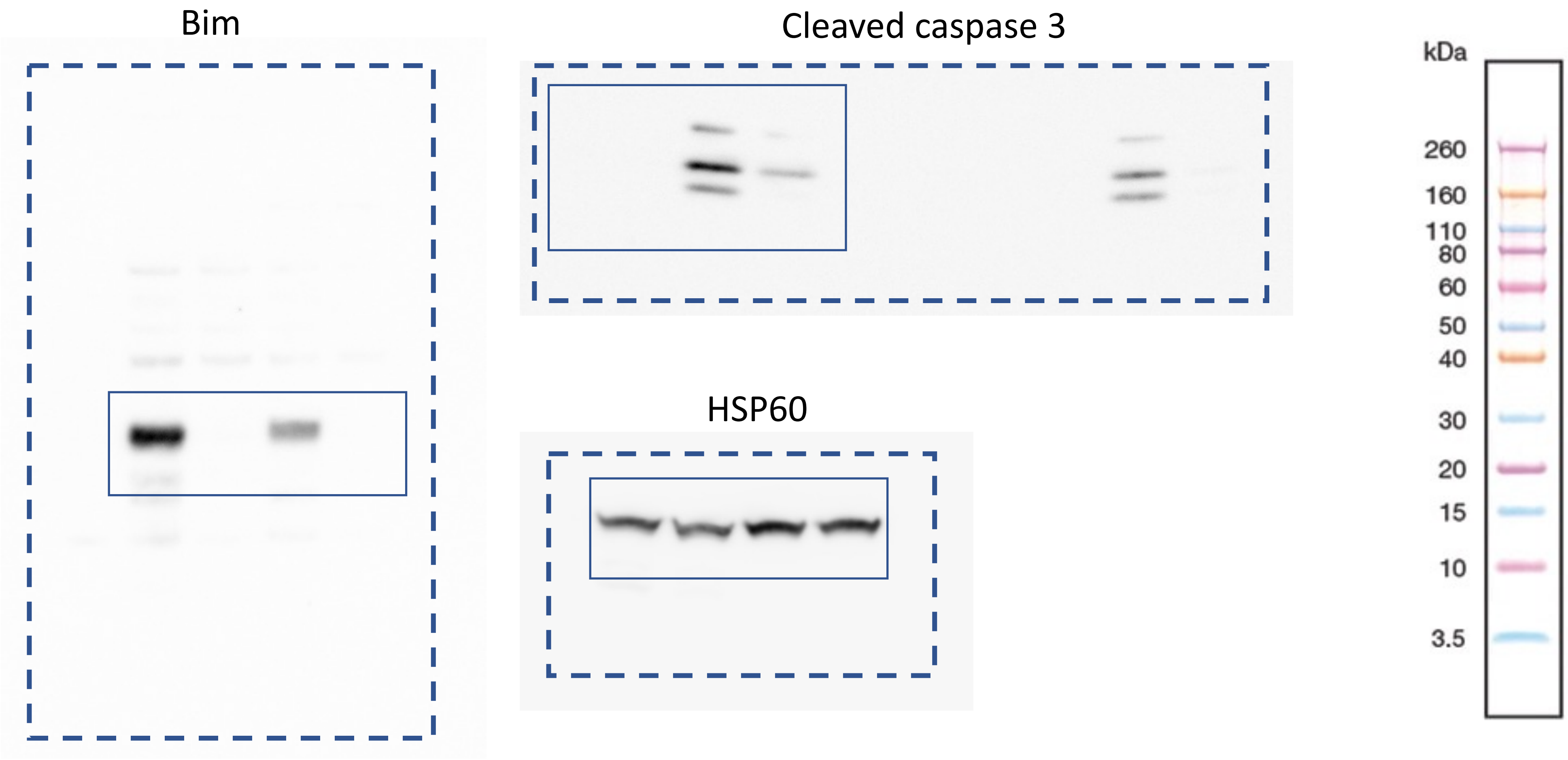

Figure 2B

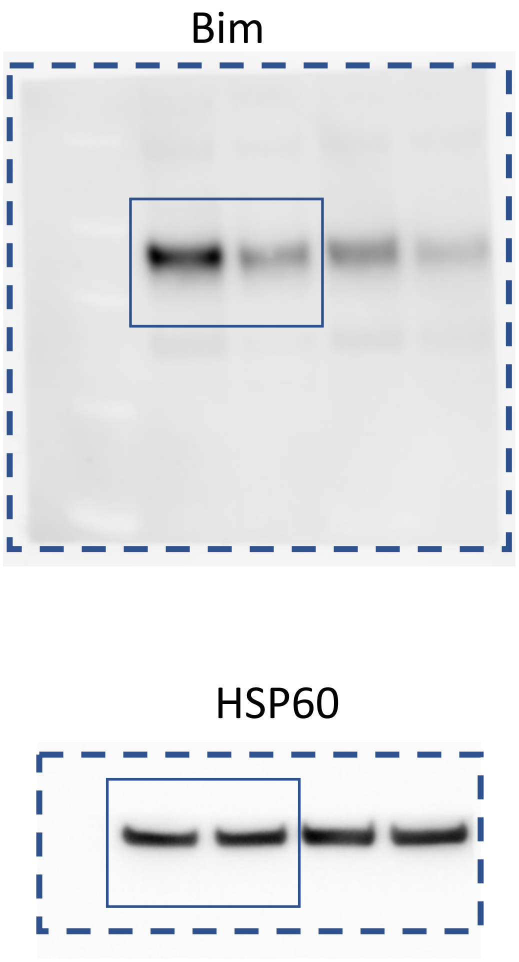

Figure 2C

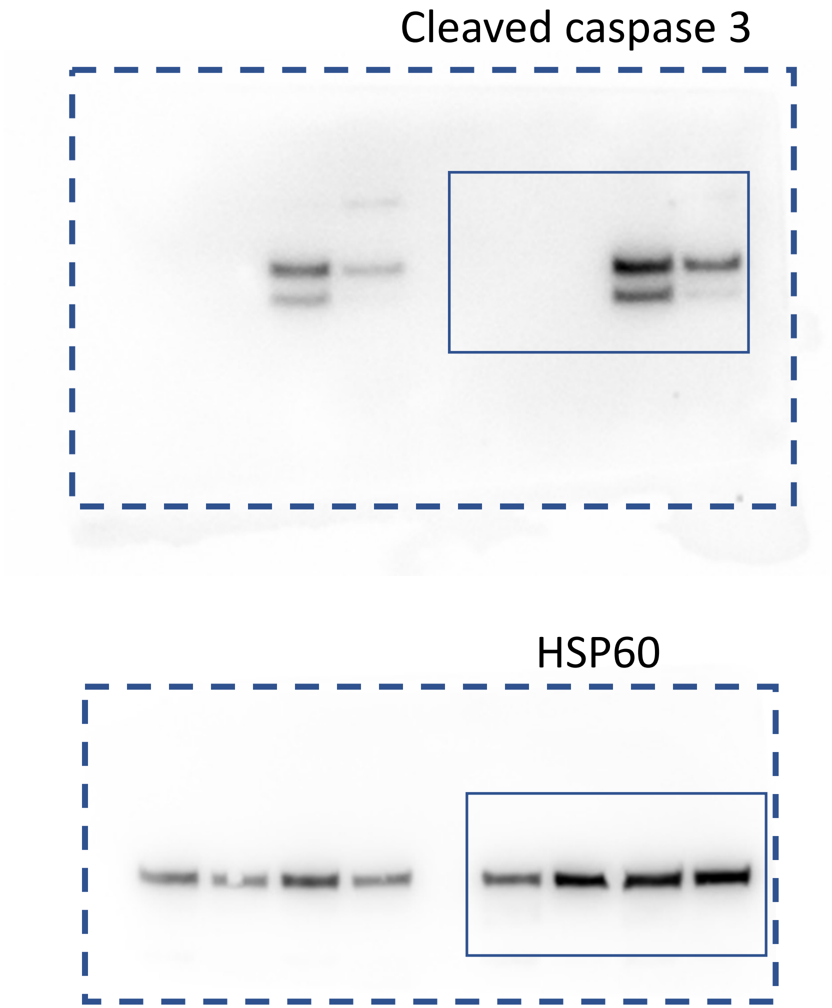

Figure 2D

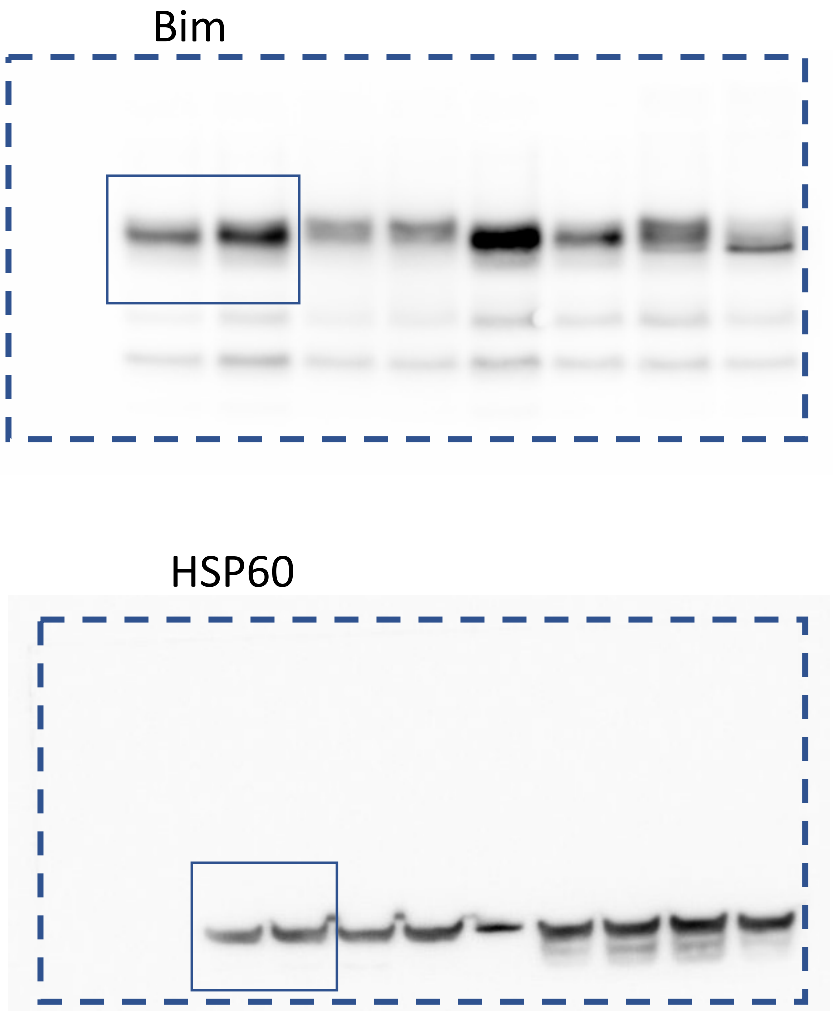

Fig. 2E

Cleaved caspase 3

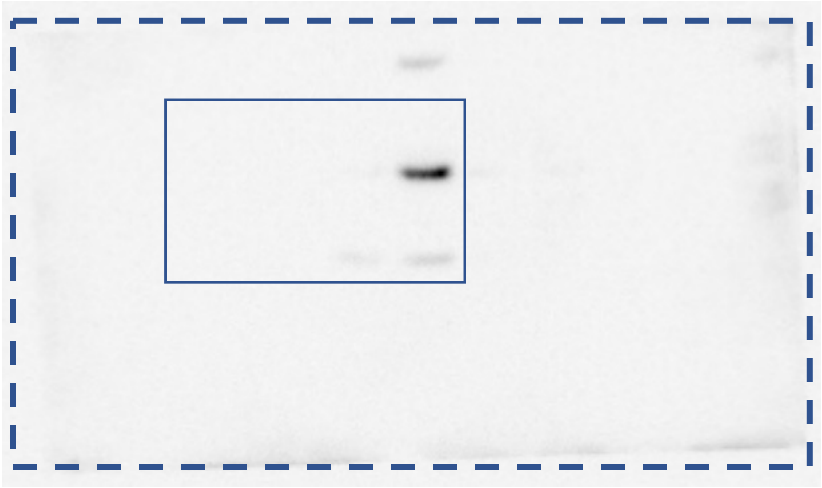

HSP60

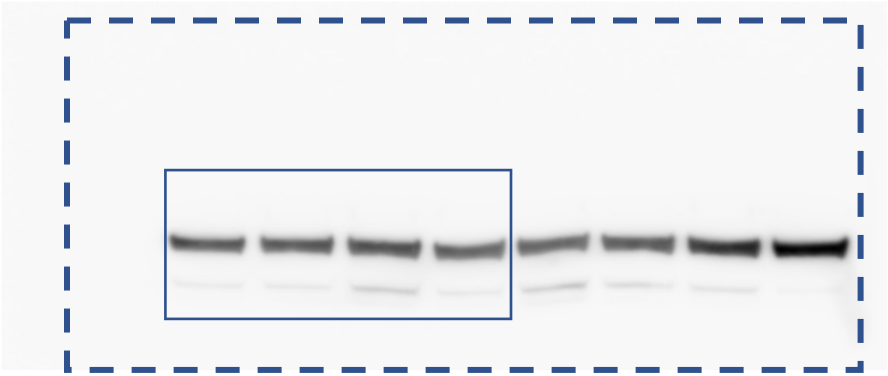

Figure 2L

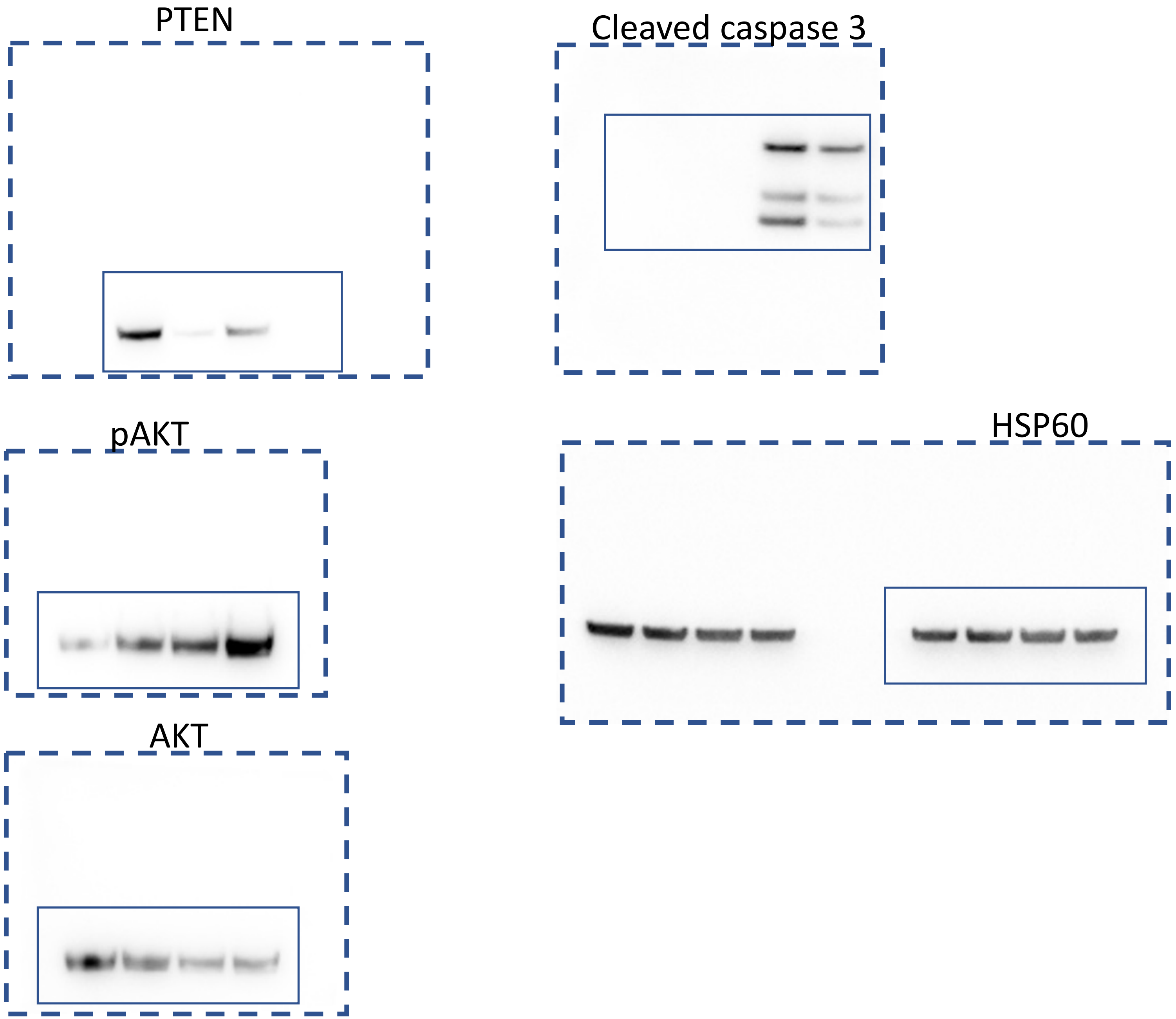

Figure 2M

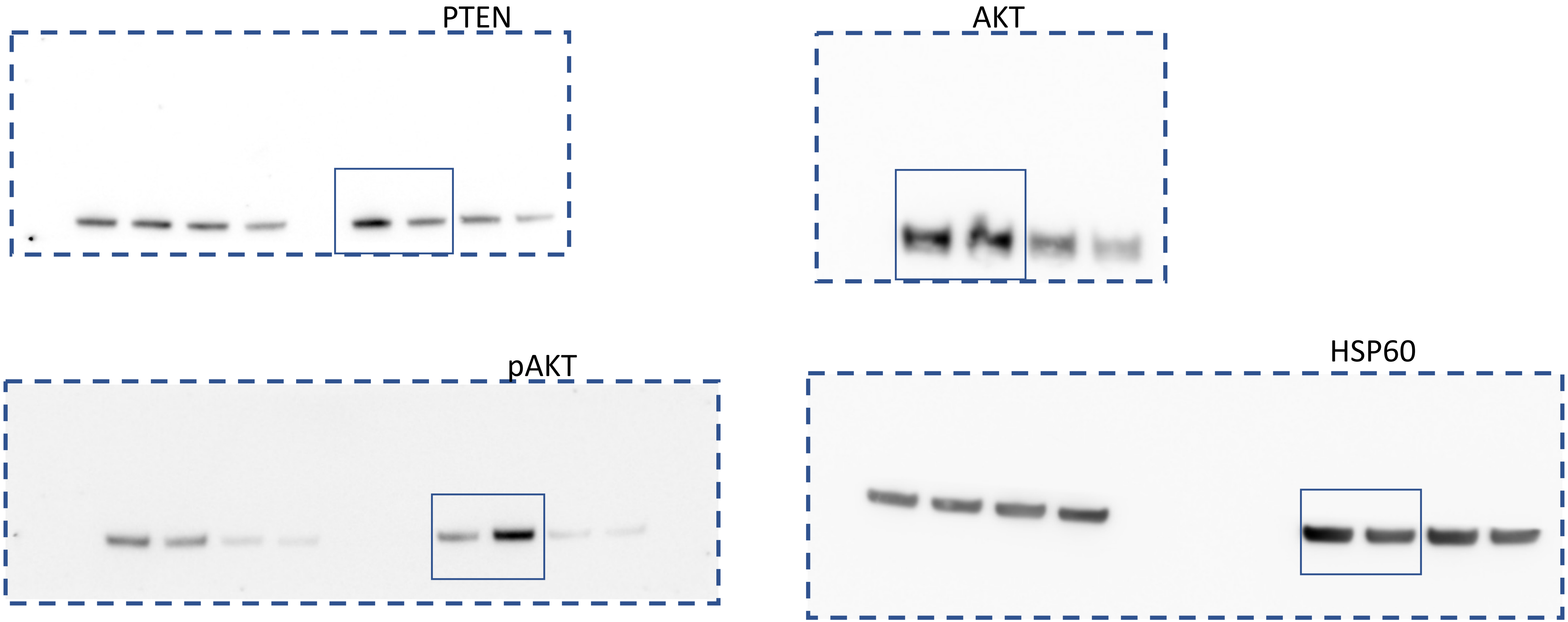

Figure 2N

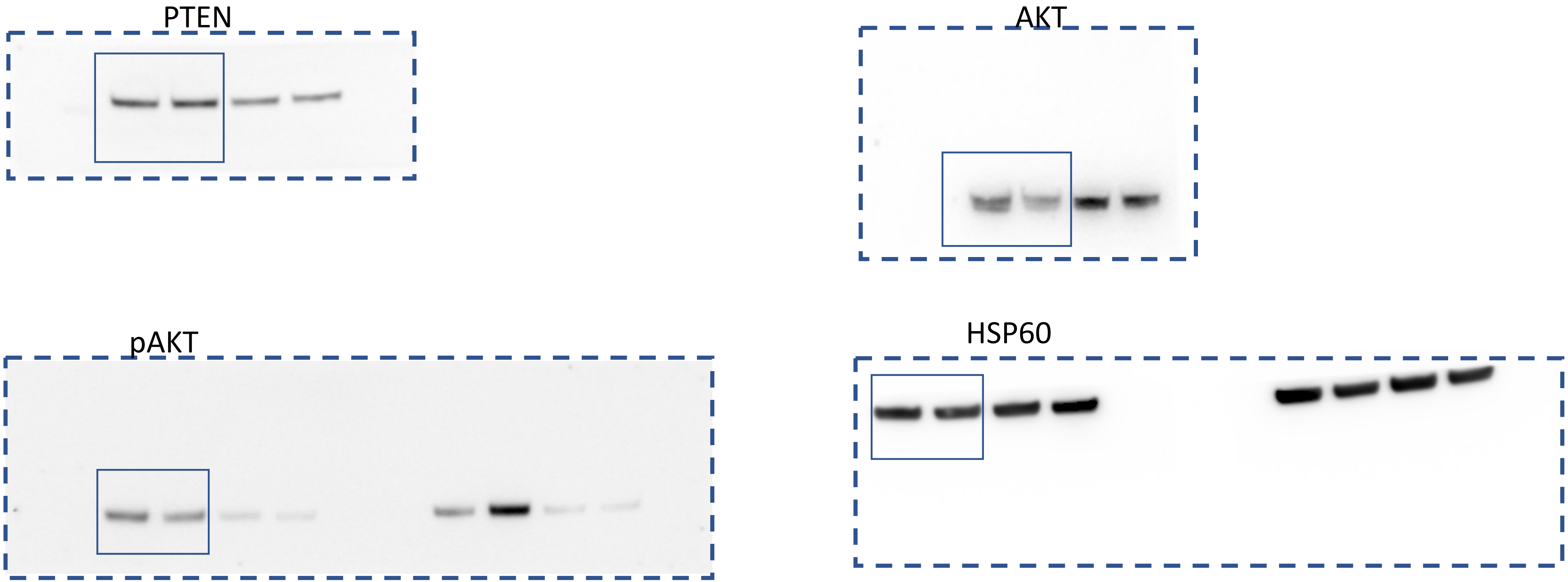

Figure 3A

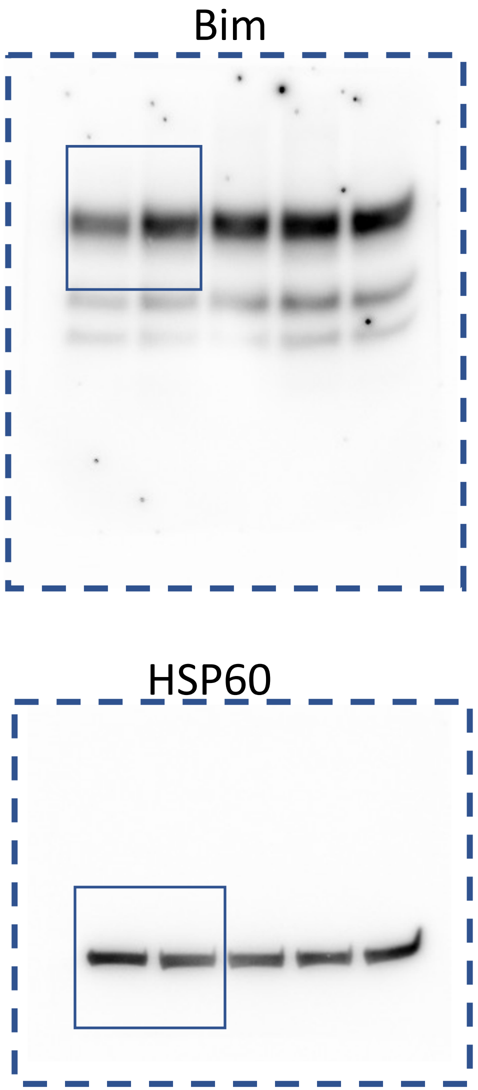

Figure 3B

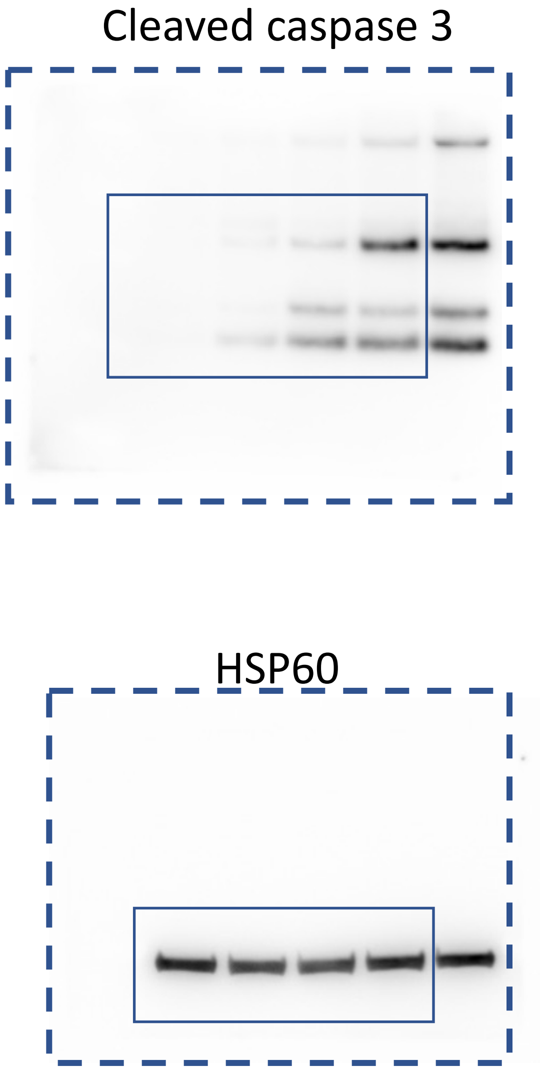

Figure 3C

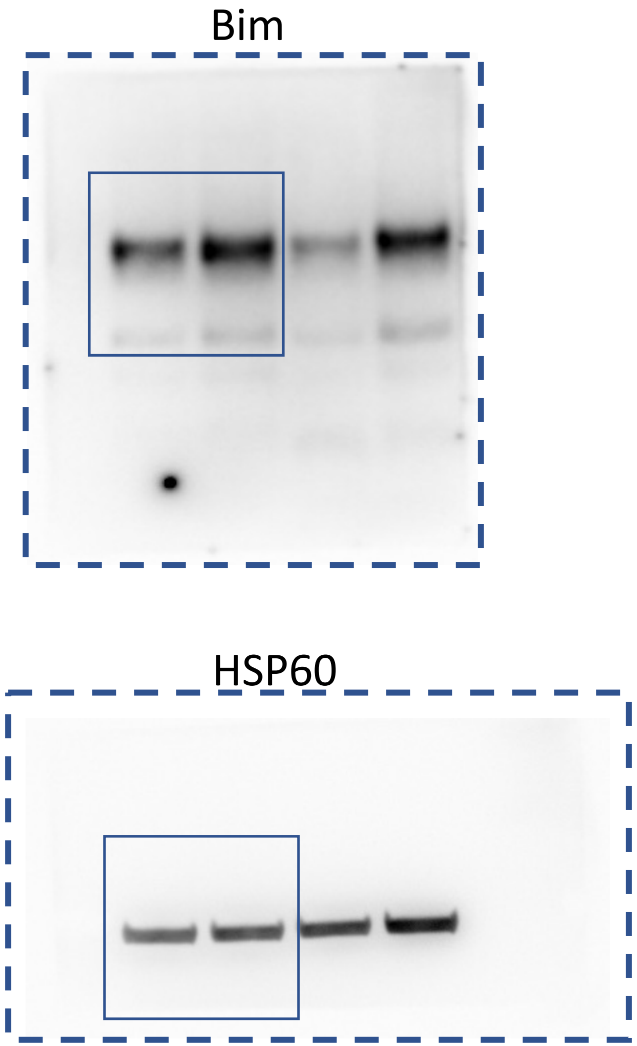

Figure 3D

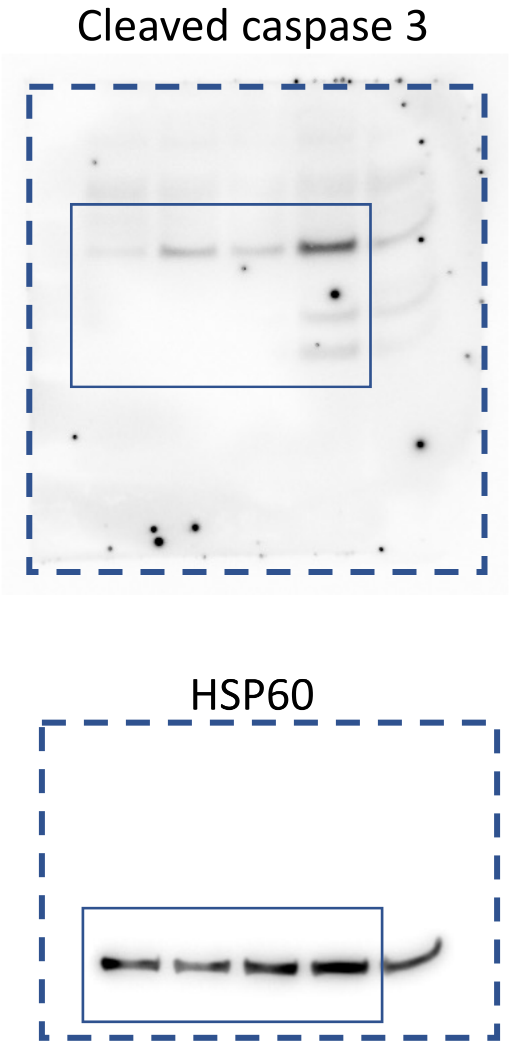

Figure 5A

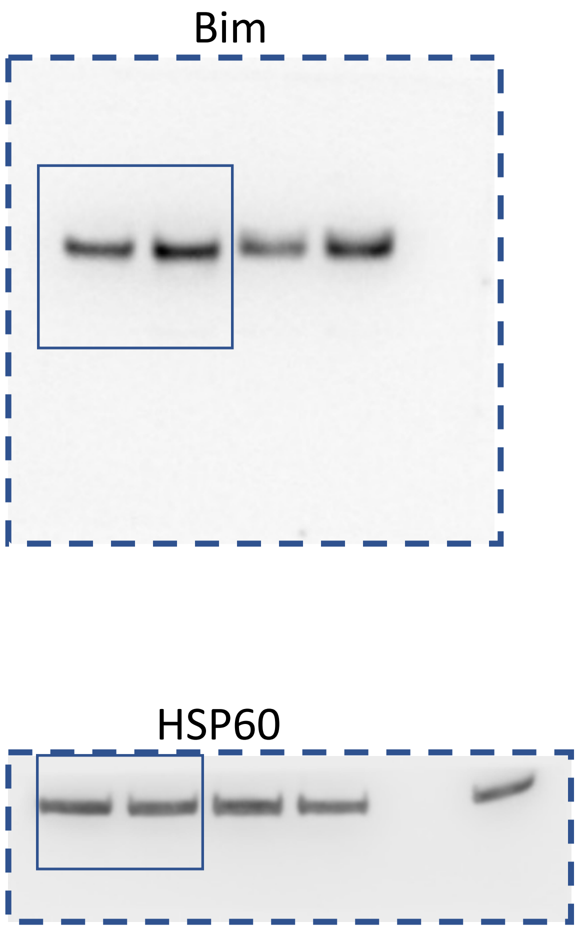

Figure 5B

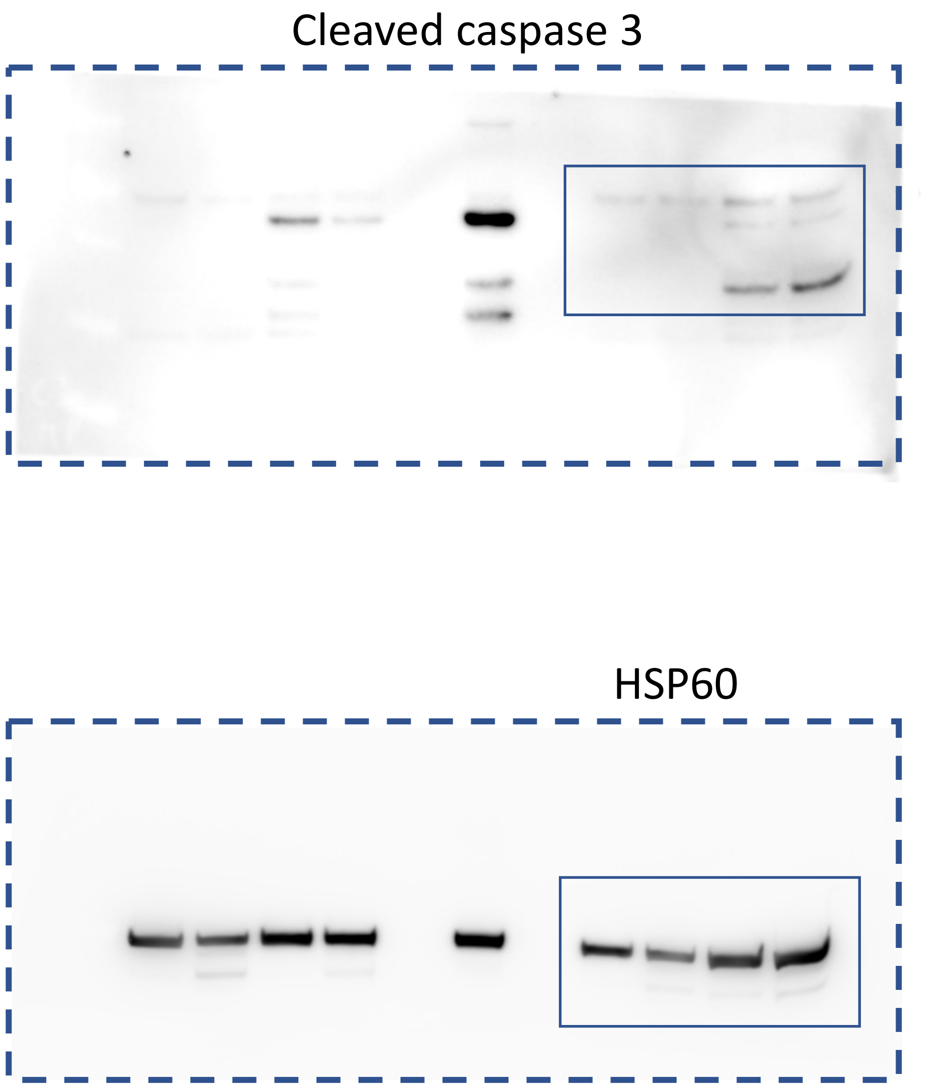

Figure S1

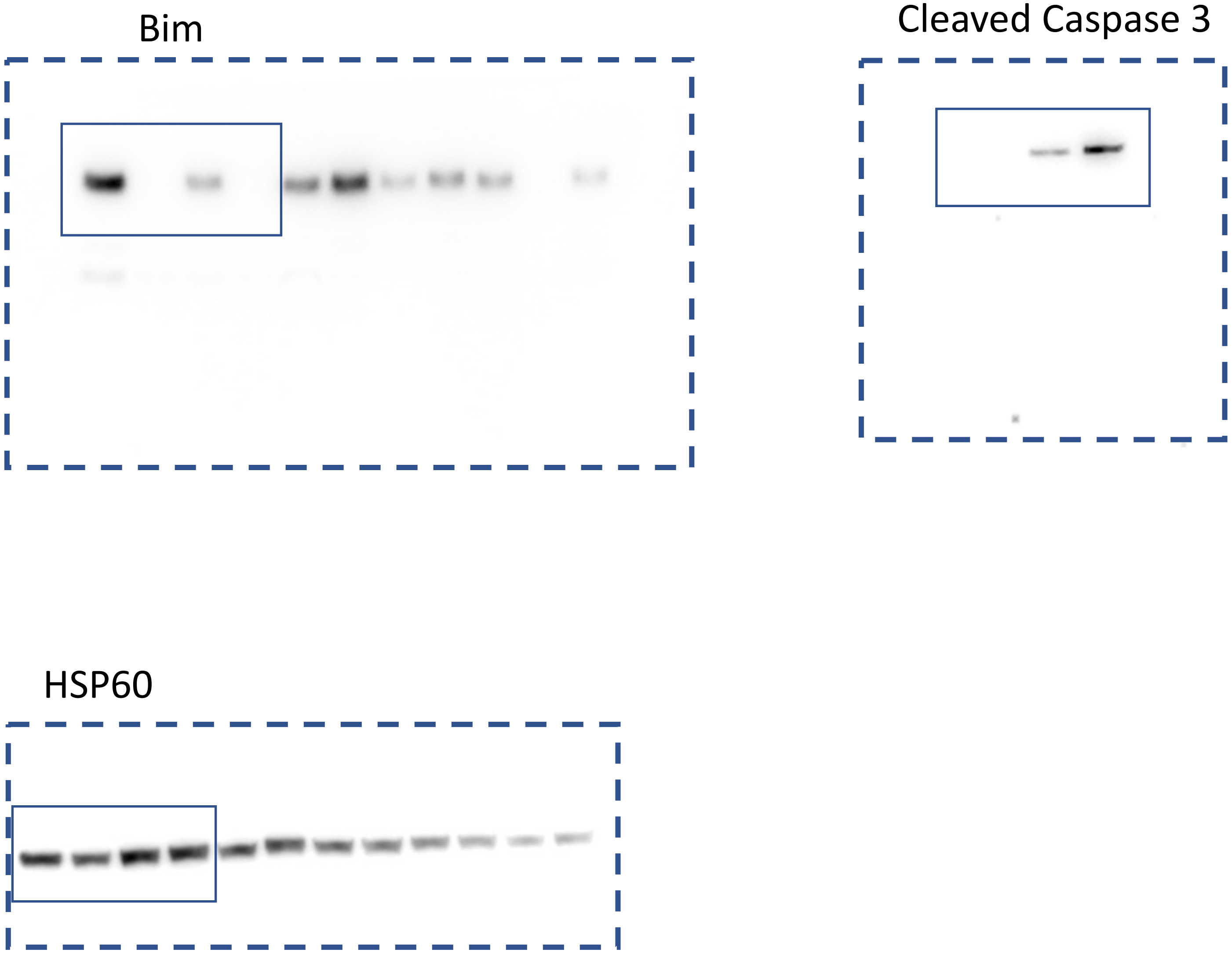

Figure S2D

Bim

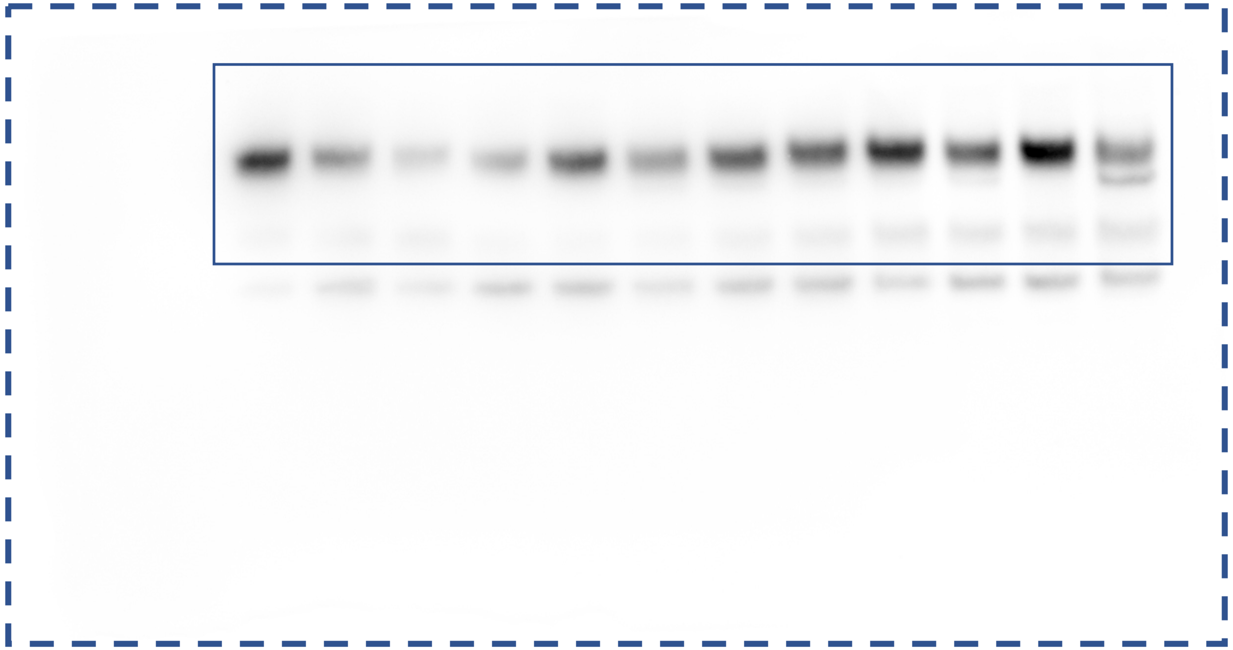

Cleaved Caspase 3

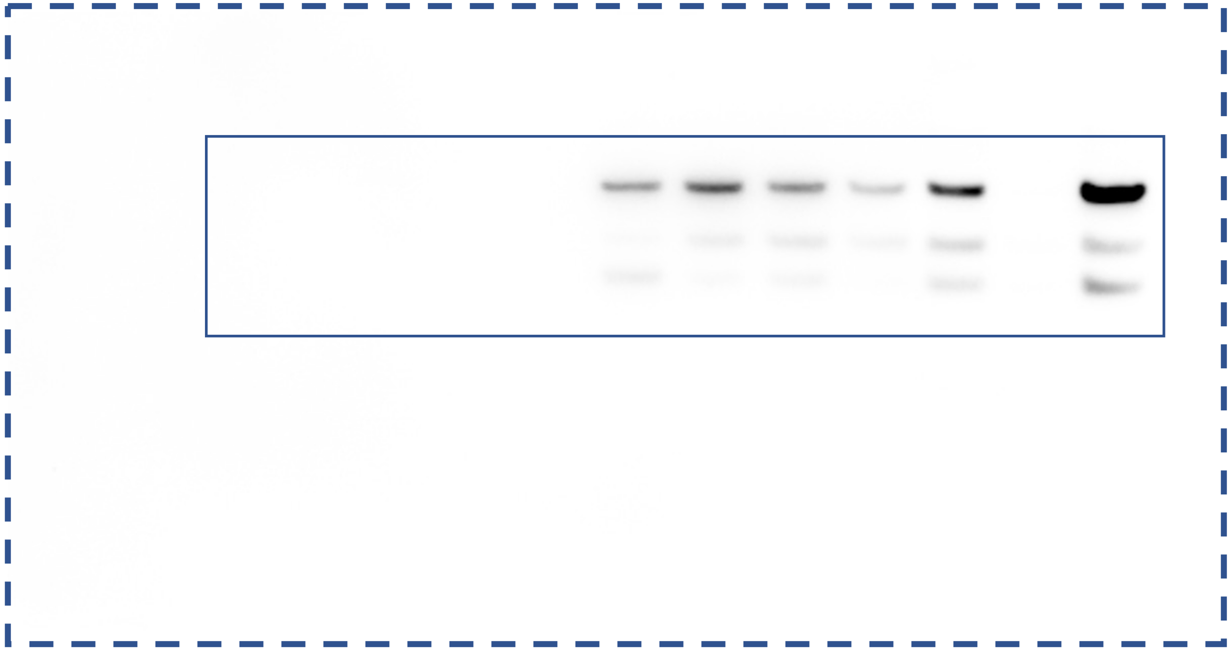

HSP60

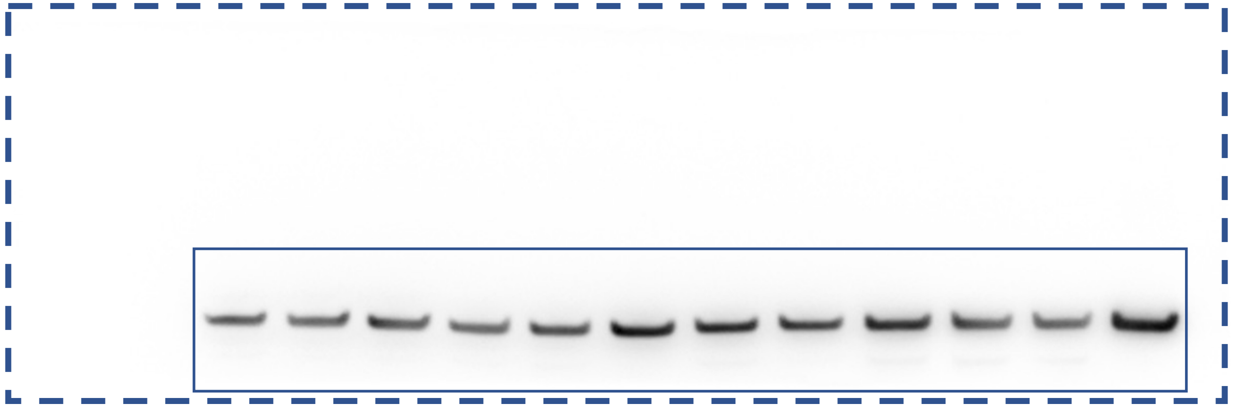

Supplement: Supplementary file 2 — Uncropped WB [file 41419_2023_6125_MOESM2_ESM.pdf]
