# Supplementary material for: miR-92a-3p regulates cisplatin-induced cancer cell death
Source: Cell Death Dis. 2023 Sep 13;14(9):603. doi: 10.1038/s41419-023-06125-z (PMC10499794; doi:10.1038/s41419-023-06125-z)
Supplement: Supplementary file 4 — Autorship change approval [file 41419_2023_6125_MOESM4_ESM.pdf]

Manuscript "miR-92a-3p regulates cisplatin-induced cancer cell death" by Cynthia Van der Hauwaert, Romain Larrue, Sandy Fellah, Nihad Boukrout, Corentin De Sousa, Julie Lemaire, Carolane Leboeuf, Marine Goujon, Michael Perrais, Bernard Mari, Christelle Cauffiez, and Nicolas Pottier [Paper #CDDIS-23-1179RR]

- 1st author : Romain Larrue

De: "LARRUE Romain" <Romain.LARRUE@chu-lille.fr>
À: "Nicolas Pottier" <nicolas.pottier@univ-lille.fr>
Cc: "cynthia vanderhauwaert" <cynthia.vanderhauwaert@inserm.fr>
Envoyé: Lundi 28 Août 2023 04:04:56
Objet: RE: Article CDD

Ok pour Bernard Mari.

- 2nd author : Sandy Fellah

From: Sandy Fellah <sandy.fellah@hotmail.fr>
Sent: Saturday, August 26, 2023 11:23:41 AM
To: cynthia vanderhauwaert <cynthia.vanderhauwaert@inserm.fr>
Cc: pottier nicolas <nico_pottier@yahoo.fr>
Subject: Article CDD

Bonjour Cynthia et Nicolas,

Ok pour Bernard Mari

Bon week-end
Sandy

- 3rd author : Nihad Boukrout

----- Mail original -----
De: "nihad boukrout" <nihad.boukrout@inserm.fr>
À: "cynthia vanderhauwaert" <cynthia.vanderhauwaert@inserm.fr>
Cc: "Nicolas Pottier" <nicolas.pottier@univ-lille.fr>
Envoyé: Lundi 28 Août 2023 06:22:03
Objet: Article Cddisease

Bonjour Cynthia,

Ok pour Bernard Mari

Bonne journée,

Cordialement,

Nihad Boukrout

- 4th author : Corentin De Sousa

De: "corentin desousa59115" <corentin.desousa59115@gmail.com>
À: "cynthia vanderhauwaert" <cynthia.vanderhauwaert@inserm.fr>, "Nicolas Pottier" <nicolas.pottier@univ-lille.fr>
Envoyé: Samedi 26 Août 2023 13:16:57
Objet: Article CDD

OK pour Bernard

- 5th author : Julie Lemaire

De: "Julie LEMAIRE" <lemairejulie32@gmail.com>
À: "cynthia vanderhauwaert" <cynthia.vanderhauwaert@inserm.fr>
Cc: "Nicolas Pottier" <nicolas.pottier@univ-lille.fr>
Envoyé: Samedi 26 Août 2023 15:38:03
Objet: Article CDD

Ok pour bernard mari

- 6th author : Carolane Leboeuf

De: "Carolane Leboeuf" <carolaneleboeuf@outlook.com>
À: "cynthia vanderhauwaert" <cynthia.vanderhauwaert@inserm.fr>
Cc: "Nicolas Pottier" <nicolas.pottier@univ-lille.fr>
Envoyé: Samedi 26 Août 2023 15:46:19
Objet: Article CDD

Bonjour,

Ok pour bernard mari.

- 7th author : Marine Goujon

----- Mail original -----
De: "Marine Goujon" <marine.goujon@univ-lille.fr>
À: "cynthia vanderhauwaert" <cynthia.vanderhauwaert@inserm.fr>
Cc: "Nicolas Pottier" <nicolas.pottier@univ-lille.fr>
Envoyé: Lundi 28 Août 2023 03:28:06
Objet: Article

Ok pour bernard mari

- 8th author : Michael Perrais

De: "michael perrais" <michael.perrais@inserm.fr>
À: "cynthia vanderhauwaert" <cynthia.vanderhauwaert@inserm.fr>
Cc: "Nicolas Pottier" <nicolas.pottier@univ-lille.fr>
Envoyé: Samedi 26 Août 2023 15:01:59

OK pour Bernard Mari.

- 9th author : Bernard Mari

From: Bernard Mari <Bernard.Mari@unice.fr>
Sent: Sunday, August 27, 2023 7:13:52 PM
To: Cynthia Van Der Hauwaert <cynthia.vanderhauwaert@inserm
Cc: nicolas pottier <nico_pottier@yahoo.fr>
Subject: Re: Fwd: CDDIS-23-1179RRR Initial Quality Check

Dear Cynthia,

I approve the change regarding the new author list.

Best regards,

Bernard Mari, Ph.D
Institut de Pharmacologie Moléculaire et Cellulaire (IPMC)
UMR7275 CNRS - Université Côte d'Azur
660, Route des Lucioles
F-06560 Sophia Antipolis
FRANCE
Tel : +33 4 93 95 77 19
email: Bernard.MARI@univ-cotedazur.fr

- <u>10th author : Christelle Cauffiez</u>

De: "Christelle Cauffiez" <christelle.cauffiez@univ-lille.fr>
À: "cynthia vanderhauwaert" <cynthia.vanderhauwaert@inserm.fr>
Cc: "Nicolas Pottier" <nicolas.pottier@univ-lille.fr>
Envoyé: Samedi 26 Août 2023 13:54:45
Objet: Fwd: Article CDD

OK for Bernard Mari authorship

Christelle Cauffiez

- <u>11th author : Nicolas Pottier</u>

De: "Nicolas Pottier" <nicolas.pottier@univ-lille.fr>
À: "cynthia vanderhauwaert" <cynthia.vanderhauwaert@inserm.fr>
Cc: "Nicolas Pottier" <nicolas.pottier@univ-lille.fr>
Envoyé: Samedi 26 Août 2023 17:17:15
Objet: CDD

Ok pour Bernard Mari

- <u>12th author : Cynthia Van der Hauwaert</u>

De: "cynthia vanderhauwaert" <cynthia.vanderhauwaert@inserm.fr>
À: "Nicolas Pottier" <nicolas.pottier@univ-lille.fr>
Envoyé: Samedi 26 Août 2023 18:53:50
Objet: RE: CDD

ok pour Bernard Mari

Dr Cynthia Van der Hauwaert
